# Antennal Sensilla Diversity in Some North American Cicadas (Hemiptera: Cicadidae)

**DOI:** 10.3390/insects17010115

**Published:** 2026-01-20

**Authors:** Allen F. Sanborn

**Affiliations:** 1 Department of Biology, Barry University, 11300 NE Second Avenue, Miami Shores, FL 33161-6695, USA; asanborn@barry.edu; 2 Department of Physiology and Biophysics, University of Illinois at Urbana-Champaign, 524 Burrill Hall 407 S. Goodwin Ave., Urbana, IL 61801, USA

**Keywords:** cicada, antenna, morphology, SEM, Fidicinini, Tacuini, Leptopsaltriini, Lamotialnini, Tibicinini, Platypediini

## Abstract

The use of acoustic communication and the relatively small antennae possessed by cicadas has led to the suggestion that antennal functions, specifically olfaction, are poorly developed in cicadas. The first step in determining antennal functions is to investigate the fine antennal structure. Scanning electron microscopy was used to visualize the morphology of the antennal sensilla in a diverse group of North American cicadas. The sensilla types and their distribution on the antennae of 30 species representing 12 genera, 6 tribes, and 3 subfamilies of North American cicadas are described and/or illustrated. Seven main classes and several subclasses of antennal sensilla were found: sensilla trichodea, sensilla chaetica, sensilla coeloconica, sensilla styloconica, foramina olfactoria, sensilla campaniformia, and sensilla cavitata-peg. Unique sensilla types and/or organizations of sensilla were found in many of the genera and differences between species of some genera were also present. No sexual dimorphism within species was found in the types or organizational patterns of the sensilla. The diversity of sensilla provides additional data for taxonomy and phylogenetic analyses. The potential function of the various sensilla types is hypothesized. Although relatively small, the antennae of cicadas are likely to provide significant information about their environment and increase their survival, including the selection of specific host plants and microhabitat selection observed in many species.

## Simple Summary

Cicadas are known for the songs produced in the mating process. Combining the acoustic behavior with relatively small antennae has meant that antennal sensory functions, specifically olfaction, have been suggested to be poorly developed in cicadas. The selection of specific host plants by many species suggests that the antennae are important in finding these host plants. Scanning electron microscopy was used to visualize the morphology of antennal sensilla found in a diverse group of North American cicadas. The sensilla types and the distribution pattern of each sensillum type found on 30 species from 12 genera, 6 tribes, and 3 subfamilies of North American cicadas are described and/or illustrated. Unique sensilla types and/or organizations of sensilla were found in many of the genera. This diversity provides additional data for taxonomy and phylogenetic analyses. The potential function of the various sensilla types is hypothesized. Although relatively small, the antennae of cicadas are likely to provide significant information about their environment and increase their ability to survive and reproduce successfully.

## 1. Introduction

Cicadas are associated with the sounds of summer. The mating calls are an obvious feature of cicada biology, and much work has gone into the study and analysis of sound production and reception, e.g., [1,2]. Study of other sensory modalities has been extremely limited.

Anatomy and physiology are interdependent in animals. Cicada anatomy, particularly in male specimens, has been highly modified to increase the efficiency of sound production. In contrast, the antennae of cicada are small and exhibit a relatively simple construction. Cicada antennae have been analyzed morphologically only a few times [3,4,5]. Cicada antennae are composed of a scape, pedicel, and five flagellar segments. The flagellar segments generally taper distally, except in the South American genus *Mendozana* Distant, 1906, whose distal antennae can expand into a broad plate [6].

The use of acoustic communication and the relatively small antennae possessed by cicadas has led to the suggestion that antennal functions, specifically olfaction, are rudimentary in the “poorly developed” [7] antennae of cicadas. Myers [7] ranked the importance of the senses in cicadas, starting with visual and auditory senses followed by tactile and finally olfactory sensation. However, there are observations that olfaction is an important sense in cicadas. Many cicada species are host-plant-specific, e.g., [8]. It has been suggested that species are able to share habitats because they select different host plants [9], a process probably directed through antennal olfaction.

Studying the morphology of antennae can provide clues as to the functions they serve. This investigation was performed as the first step to decipher the apparent function of the antennae in a phylogenetically diverse group of North American cicadas. The unique types and distribution of receptors found in some taxa provide opportunities to investigate phylogenetic trends in the antennal morphology. Comparative analysis of cicadas from Asia [4,5] provides the opportunity to investigate convergent patterns in multiple evolutionary lineages of cicadas.

## 2. Materials and Methods

Thirty species representing 12 genera, 6 tribes, and 3 subfamilies of Cicadidae Batsch, 1789 were studied (Table 1). The specimens used in this study are deposited in the author’s collection. The higher taxonomy follows Marshall et al. [10] and Dmitriev and Sanborn [11], with new names or generic assignments incorporated from Dmitriev [12] and Cole et al. [13]. The current cicada taxonomy can be viewed at the World Auchenorrhyncha Database (https://hoppers.speciesfile.org/otus/5973/overview (accessed on 1 January 2026)) [14].

Specimens for scanning electron microscopic analysis were prepared by one of two methods. The initial specimens used in the microscope were obtained from a dry mounted collection. The specimens were air-dried on pins or were originally preserved in 70% alcohol in the field and dry-mounted on pins for permanent storage. Once the viability of the process was confirmed, a procedure that would permit additional microscopic techniques was utilized. Live specimens were decapitated into a vial containing 4% glutaraldehyde in 0.1 M phosphate buffer, pH = 7.2 (Sörensens formula [16]), and stored under cold conditions until further processing could occur in the lab, a process that has been shown to retain fine structure in tissues for periods of several months [17]. Processing continued with an overnight rinse in 0.1 M phosphate buffer and post-fixing overnight in the refrigerator with a 1% osmium tetroxide in 0.1 M phosphate buffer solution. Specimens were rinsed twice with distilled water for 15 min each, then dehydrated through a graded ethanol series (10%, 25%, 50%, 75%, 85%, 95%, and three times at 100%) using 15 min washings. No apparent differences were found in the specimens based on the method of preparation prior to mounting of specimens for the electron microscope.

Heads, or head and thoracic segments in smaller species, were mounted on 12.7 mm diameter, 3.2 mm peg-lifting grooved aluminum SEM stubs (M.E. Taylor Engineering, Inc., Rockville, MD, USA) with TV Tube Koat (Ted Pella, Inc., Redding, CA, USA) carbon paint. The specimens were then sputter-coated with gold/palladium (60:40) at 40 mA in a SPI Sputter sputter coater (SPI Supplies, West Chester, PA, USA). The total amount of time the specimens were coated was determined by the degree of charging encountered after the initial coating procedure.

The International Scientific Instruments, Inc. model DS-130 Scanning Electron Microscope in the Center for Electron Microscopy at the University of Illinois at Urbana Champaign was used to view and image the specimens. An accelerating voltage of 10 kV was used to image the specimens. Micrographs were taken on Polaroid film type PN-55 (Polaroid America, New York, NY, USA, providing a picture and negative of the image produced in the microscope.

The terminology for naming structures follows Snodgrass [18], Schneider [19], Schneider and Steinbrecht [20], Callahan [21], Altner and Prillinger [22], Zacharuck [23,24], Chapman [25], and Keil [26]. Comparisons with the previously published work on cicada antennae [3,4,5] are also included.

No generative artificial intelligence was used in the production of this work.

## 3. Results

### 3.1. Antennal Structures of North American Cicadas

**Figure 1:**
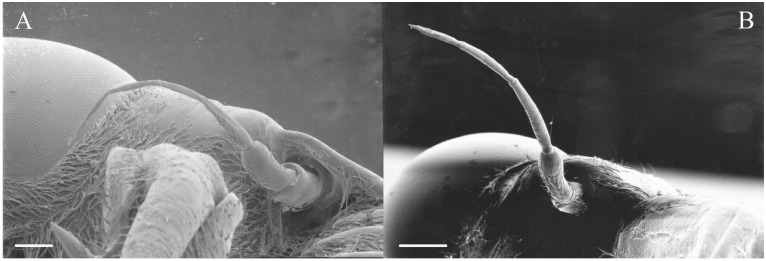
Examples of the setaceous antennae of cicadas. Trichoid sensilla are concentrated on the scape and pedicel and the other receptor types are concentrated primarily on the first flagellar segment. (**A**) *Dicerocprocta apache*. (**B**) *Magicicada septendecula*. Scale bars: (**A**) = 300 μm, (**B**) = 500 μm.

Cicada antennae are setaceous or setiform antennae, bristle-like with segments decreasing in size distally (Figure 1). The antennae are composed of a scape, a pedicel, and five flagellar segments, with the distal flagellar segments often shriveled [3,5]. The distal segments did not survive the preparation process for electron microscopy in some specimens. Seven main classes and several subclasses of antennal sensilla were found within the 30 species examined: sensilla trichodea, sensilla chaetica, sensilla coeloconica, sensilla styloconica, foramina olfactoria, sensilla campaniformia, and sensilla cavitata-peg. Unique sensilla types and/or organizations of sensilla were found in many of the genera, and differences in sensilla patterns between species of some genera were also present. No sexual dimorphism within species was found in the types or organizational patterns of the sensilla.

#### 3.1.1. Sensilla Trichodea

Sensilla trichodea are generally elongated seta with a freely moveable basal membrane. There appears to be a distinct indentation at the base of the seta suggesting that the hairs are attached to a flexible socket and are able to move. These hairs are generally mechanoreceptors but may be olfactory or gustatory if there is a terminal pore. They are found in large numbers and concentrated on the scape and pedicel in all genera examined. Trichoid sensilla are found on both dorsal and ventral surfaces, although density is usually greater on the ventral side, rather than being concentrated only on the ventral side like the remaining sensilla types. The lateral surfaces of the scape and pedicels are often devoid of sensilla and the trichoid sensilla may be restricted to the distal half of the scape. There are three subclasses of trichoid sensilla found in the North American fauna.

**Figure 2:**
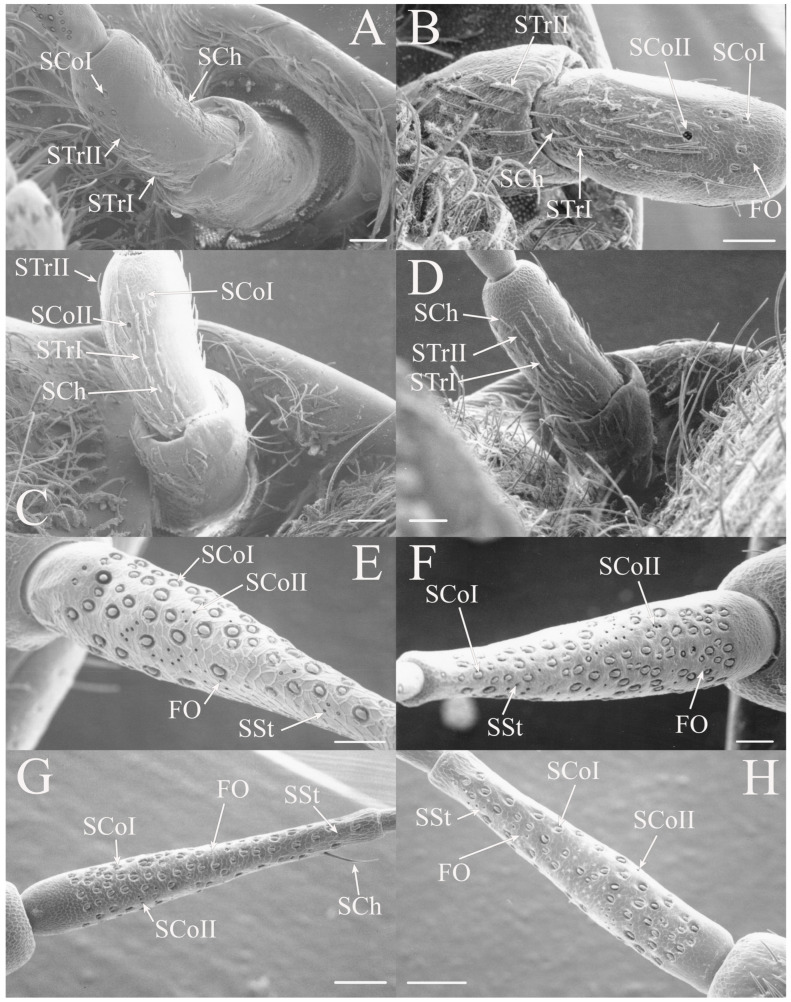
Antennal segments of *Diceroprocta* species. (**A**) Scape and pedicel of *D. apache*. Note the sensilla diversity on the pedicel, a character unique to several members of the genus. (**B**) Scape and pedicel of *D. cinctifera cinctifera*. (**C**) Scape and pedicel of *D. semicincta*. (**D**) Scape and pedicel of *D. eugraphica*. (**E**) First flagellar segment of *D. apache*. (**F**) First flagellar segment of *D. canescens*. (**G**) First flagellar segment of *D. eugraphica*. (**H**) First flagellar segment of *D. swalei swalei*. Scale bars: (**A**–**D**,**G**,**H**) = 100 μm, (**E**,**F**) = 50 μm, (**D**) = 5 μm.

STrI are long, straight seta that are generally angled distally with lengths of 53–340 μm (e.g., Figure 2B, Figure 19H, Figure 20B and Figure 22B). They are generally restricted to the scape and pedicel, with a few examples on basal flagellar segments.

STrII are shorter and generally curved with lengths of 46–168 μm (e.g., Figure 2B, Figure 19A, Figure 20B and Figure 22B). They are also angled distally but have been reported to lie in the same plane as the antennal segment [3].

STrIII are dorsoventrally flattened along their length and curved with lengths of 39–52 μm (e.g., Figure 5D,E and Figure 7C). They generally lie along the same plane as the antennal segment. They are a primary trichoid sensillum on the scape and pedicel of *Beameria*.

Klein et al. [3] and Wang et al. [5] each found three subclasses of trichoid sensilla, although one of the subclasses described by Klein et al. [3] is considered to be a sensillum chaeticum (see below). The subclasses defined by both groups are similar, although the absolute lengths provided for each subgroup are variable. This is probably due to the much greater species diversity (25 species from 22 genera) included in Wang et al.’s study [5] compared to the single species studied by Klein et al. [3].

#### 3.1.2. Sensilla Chaetica

Sensilla chaetica (SCh) are similar to sensilla trichodea but are more bristle-like or spine-like rather than being setiform. The thick walls of this type of sensilla will prevent molecules from passing to internal receptors so they appear to be primarily mechanoreceptors. This type of receptor was found associated primarily with the scape and pedicel but sensilla were found on the flagellar segments in some taxa. The sensilla chaetica found between the head and scape are generally termed Böhm’s bristles [19].

Sensilla chaetica are between 22 and 120 μm in length and distributed primarily on the scape and pedicel in the same regions where trichoid sensilla are found in the individual species (e.g., Figure 2G, Figure 5G, Figure 7C and Figure 15D). Like trichoid sensilla, the density is usually greatest on the ventral surfaces, with additional sensilla on the dorsal side, while the lateral surfaces are often devoid of sensilla chaetica. In addition, sensilla chaetica are found on the flagellar segments of some species, usually as a single sensillum near the distal end of a flagellar segment, but were also found extending from the distal end of the terminal flagellum. The smallest were found on the flagellar segments of *Hadoa* (5.5 μm) and *Beameria* (11 μm). The sensilla found on the terminal flagellar segments were generally about 50 μm in length. Böhm’s bristles were also found but were shorter, only about 22 μm in length (e.g., Figure 7A,B).

Klein et al. [3] describe a hair sensillum that is shorter and more bristle-like than the other types of hair-like sensilla they found. This sensillum should be classified as a sensillum chaeticum. A similar bristle-like sensillum chaeticum was described in the adult specimens studied by Li and Wei [4] but the sensillum was reclassified in the same species as a sensllum basiconicum by Wang et al. [5]. These structures are classified as sensilla chaetica here based on the definitions in the anatomical references [18,19,20,21,22,23,24,25,26], the well-defined socket seen in the images in both references [4,5], and the lack of numerous surface pores characteristic of sensilla basiconica [19,20].

#### 3.1.3. Sensilla Coeloconica

Sensilla coeloconica are pegs that are sunken into depressions in the antennal surface. The sensilla coeloconica can have smooth or porous surfaces which may differ in their innervation patterns and functions. This class of receptors is the major type of receptors found on the first flagellar segment in all genera, with some sensilla found on the pedicel and more distal flagellar segments in specific genera or species. There were two primary types of coeloconic sensilla observed. A potential third type may exist, but no sensillum is visible from the surface and sectioning was not performed to verify the presence of a sensillum deep within the cuticular depression as was found in *Magicicada* by Klein et al. [3].

SCoI are larger, with the majority or all of at least one side of the sensillum visible from the surface (e.g., Figure 4C, Figure 6C and Figure 10C). The sensilla are approximately 5–20 μm in length. Many are multi-porous, suggesting they are the primary olfactory sensillum on the cicada antenna. This sensilla subclass forms the grouping of coeloconic sensilla found in species of *Tibicinoides*, *Okanagana*, and *Platypedia* (e.g., Figure 18A,C, Figure 19G and Figure 22E).

**Figure 3:**
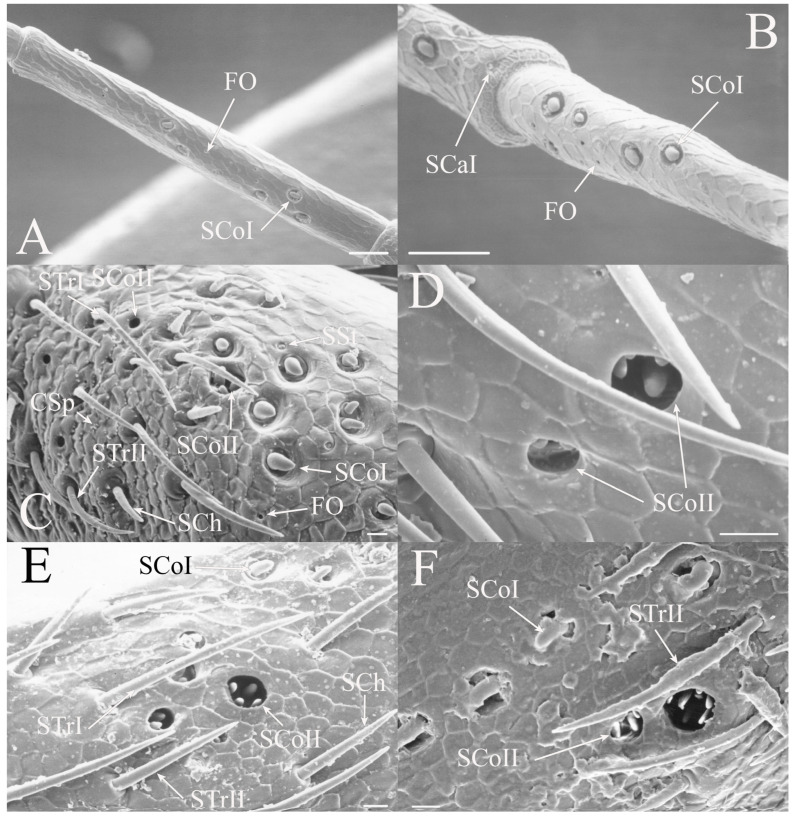
Antennal segments and magnified sensilla of *Diceroprocta* species. (**A**) Second flagellar segment of *D. swalei swalei*. (**B**) Second flagellar segment of *D. apache*. (**C**) Pedicel sensilla of *D. apache*. Note the cuticular spines. (**D**) Magnified SCoII on the pedicel of *D. apache*. Note the flattened SCoII sensilla. (**E**) Magnified pedicel sensilla of *D. semicincta*. Note the flattened SCoII sensilla. (**F**) Magnified pedicel sensilla of *D. cinctifera cinctifera*. Note the flattened SCoII sensilla. Scale bars: (**A**,**B**) = 50 μm, (**C**–**F**) = 10 μm.

SCoII are visible through a 2.5–6.5 μm pore-like opening in the antennal cuticle (e.g., Figure 6D, Figure 7F, Figure 8C, Figure 10A, Figure 18F and Figure 21A). They are smaller than SCoI, with only the distal tip potentially visible within the cuticular depression. Some species of *Diceroprocta* possess a larger version of this sense organ (e.g., Figure 2B,C and Figure 3C,D,F). There are multiple sensilla found within larger depressions with a single pore-like opening of 11–23 μm.

Coeloconic sensilla are the primary sense organs on the first flagellar segment. The sensilla density is usually greatest on the ventral surfaces but there are a few genera that have a central gap with other receptors surrounded by the coeloconic sensilla. Sensilla coeloconica were found on the second, third, and fourth flagellar segments and were found on the ventral scape in a couple of species.

Klein et al. [3] describe three different sizes of coeloconic sensilla. There are differences in the surface structure of the two larger receptors and the smallest receptors, with the first two subclasses having exposed sensilla in a cuticular depression and the third subclass having a peg hidden within a cavity opening through a small pore on the surface. Wang et al. [5] describe three subclasses as well, but all have visible sensilla within the cuticular depressions.

#### 3.1.4. Sensilla Styloconica

**Figure 4:**
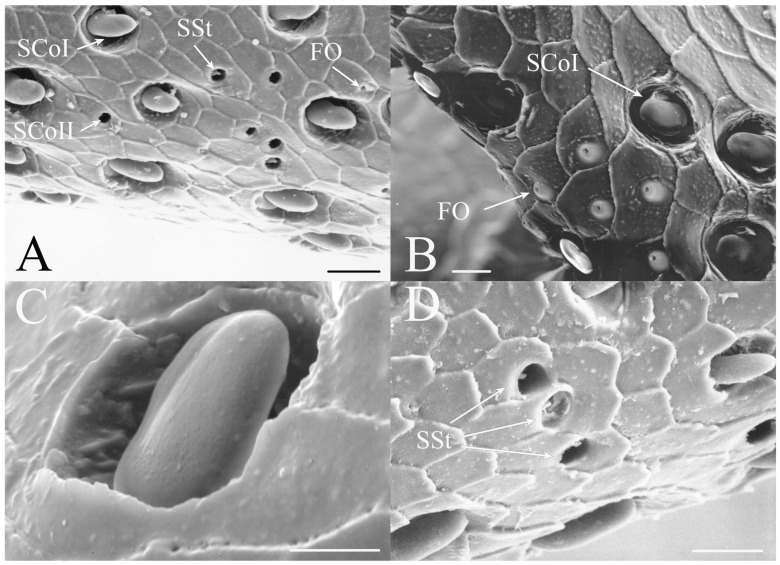
Magnified sensilla of *Diceroprocta* species. (**A**) SCoI, SCoII, SST, and FO on the first flagellar segment of *D. cinctifera cinctifera*. (**B**) SCoI and FO on the first flagellar segment of *D. apache*. (**C**) SCoI on the first flagellar segment of *D. apache*. Note the multi-porous surface. (**D**) Triplet of SSt on the first flagellar segment of *D. eugraphica*. Scale bars: (**A**,**B**,**D**) = 10 μm, (**C**) = 5 μm.

Sensilla styloconica (SSt) are similar to sensilla coeloconica but are found in deeper pits with a more restricted opening (1–5 μm) and the sensillum is cone-shaped (e.g., Figure 4A,D, Figure 6H, Figure 8C, Figure 12D and Figure 21B). The tips of what are classified as sensilla styloconica here are primarily seen within a pore-like opening on the first flagellar segment but may also be found on the pedicel or more distal flagellar segments. The tip of the sensillum is closer to the pore and the sensillum is more conical than the ovoid small coeloconic sensilla described by Klein et al. [3], whose tip cannot be seen from the surface. Wang et al. [5] describe the sensillum as a stout cone with a well-defined rim surrounding the opening to the cuticular depression containing the sensillum.

Styloconic sensilla were found on the pedicel and the three proximal flagellar segments. The greatest receptor density is on the ventral surface of the proximal flagellar segment. The sensilla were found in groups of two to five individual receptors near one another in some species. Each of the sensilla in these groups is composed of a single sensillum associated with an individual pore.

#### 3.1.5. Foramina Olfactoria

The foramina olfactoria (FO) are identified as small pores (1–4 μm) on the surface of the antennal flagella (e.g., Figure 4B and Figure 6G). These are found in specific genera and are few in number in all species if the sensillum type is present. The openings may be of similar size to SSt pores but the sensillum visible within the pore will distinguish an SSt from an FO where no sensillum is visible (e.g., Figure 18A and Figure 22E). The term has been applied only to cicadas [5] for 2–4 μm pores on the first flagellar segment and sometimes on more distal flagellar segments. The term will be employed here for similar pores where no sensillum can be seen through the pore, although these may include some of the deep coeloconic sensilla described by Klein et al. [3]. The proposed olfactory function requires experimental and cytological verification.

Foramina olfactoria were found on the pedicel and the four proximal flagellar segments. The greatest density is on the ventral surface of the proximal flagellar segment, with some species having grouped receptors within a central gap of the coeloconic sensilla. The sensilla were found individually and in groups.

#### 3.1.6. Sensilla Campaniformia

Sensilla campaniformia are generally domed structures on the surface of the antennae. The classic design is a domed structure with a central pore-like region found within a small depression on the antennal surface. A second type was also found restricted to *Magicicada*.

SCaI is the classic design of the sensillum (e.g., Figure 3B, Figure 6B and Figure 16B). They were found as 1–3.5 μm domes within 2.2–5 μm depressions. They were found on the first flagellar segment in *Beameria*, the second flagellar segment in *Diceroprocta*, and all flagellar segments in *Magicicada*. They have been reported on the scapes and pedicels of *Magicicada* [3] and apical flagellar segments in some Asian species [5].

SCaII is a second morphological type that appears as a cup or vacant hair follicle with a raised rim (e.g., Figure 14E and Figure 16C). These were generally found on the scapes and pedicels in *Magicicada* species, with additional examples found on the first flagellar segment. The sensilla appear as round or ovoid elevations with a distinct bulge on one side found on the antennal surface about 8–13 μm in diameter with an opening of about 2.5–6 μm. No sensillum can be seen within the central depression, suggesting the receptor is a deep rather than surface receptor. The distal side of the elevation is usually sunken more than the proximal side so that the opening is angled distally. They are thought to be air pressure or cuticular stress receptors.

Classic sensilla campaniformia were found on the scape, pedicel and the proximal flagellar segment. Klein et al. [3] found these on the distal regions of the first two antennal flagella. The sensilla were found individually and grouped with other types of receptors. The SCaII were found only on the scapes, pedicels, and first flagellar segments of *Magicicada* species.

Klein et al. [3] describe two classic campaniform sensilla differing in the size of the sensory cap, one with a cap of about 5 μm and one with a cap diameter of 1–2 μm, each within a slight depression, on the scape and pedicel of *M. cassinii*. Similar cuticular domes of 5.5–8.5 μm diameter were found in Asian cicadas [4,5] but restricted to the distal flagellar segments. The rimmed pores (e.g., Figure 14H and Figure 16D) of Klein et al. [3] are structurally distinct from the campaniform sensilla described above, with the rims being circular, thinner, and lacking the bulge on one side found in the SCaII.

#### 3.1.7. Sensilla Cavitata-Peg

Sensilla cavitata-peg (SCav) was first reported in some Asian cicadas by Wang et al. [5]. The sensilla are cone-like structures within a cuticular depression that may be found as a single sensillum or a group of sensilla. A similar structure to the subclass I of Wang et al. [5] was found at the base of a sensillum chaeticum on the pedicel of *Beameria* (e.g., Figure 7C,D). A grouping of about 12 pegs was found in a single crescent-moon-shaped cuticular depression at the base of a sensillum chaeticum. The opening on the antennal surface is 8 μm long and 4 μm wide at its widest point. There is a slightly raised rim on the outer curvature of the depression. The function has been suggested to be either chemoreception, mechanoreception, thermoreception, or hygroreception [5].

A single group of sensilla cavitata-peg was found at the base of a sensillum chaeticum on the pedicel of *Beameria venosa*. The distinct shape of the opening at the base of the sensillum chaeticum suggests that it is not a developmental artifact, and the group of structures forms a potential sensory organ, as described by Wang et al. [5].

### 3.2. The Antennae and Types of Sensilla Found in Different North American Taxa

#### 3.2.1. Antennal Sensilla in Species of *Diceroprocta* Stål, 1870

Six species, *Diceroporcta apache* (Davis, 1921), *D. canescens* Davis, 1935, *D. cinctifera cinctifera* (Uhler, 1892), *D. eugraphica* (Davis, 1916), *D. semicincta* (Davis, 1925), and *D. swalei swalei* (Distant, 1904), were examined.

The antennae of *Diceroprocta* species possess a scape and pedicel containing primarily STrI (58–123 μm), STrII (55–70 μm), and SCh (22–53 μm) on the ventral surface and, to a lesser extent, the dorsal surface (Figure 2, Figure 3 and Figure 4). However, a unique form of SCoII was found on the pedicel of three species with numerous, flattened sensilla found within a pocket with a single wide opening (Figure 2B,C and Figure 3C,F). There are several individual SCoI, a pair of SCoI within a single depression, and three SCoI sensilla within a deeper depression, along with a few SCoII, including a group of two and a group of four SSt and FO, SSt and FO, observed through a single 17.5 μm pore, on the ventral surface of the pedicel in *D. apache* (Figure 2A and Figure 3C,D). Cuticular spines (1–3 μm) are also visible on the pedicel of *D. apache* (Figure 3C). Similarly, there are multiple SCoI and a single large SCoII, with four sensilla visible through the 23 μm opening in *D. cinctifera cinctifera* (Figure 2B and Figure 3F). *Diceroprocta semicincta* also has multiple SCoI, SSt, FO, and a grouping of three large pores ranging in size from 11 to 16 μm containing two to four SCoII sensilla (Figure 2C and Figure 3E). The grouped SCoI sensilla on the pedicel show a unique morphology, some of the sensilla are laterally flattened. The SCoI were absent on the pedicel of *D. canescens* but were the only non-hair sensillum present on the pedicel of *D. eugraphica* (Figure 2D). *Diceroprocta swalei swalei* was unique in the genus in only having STr1, STrII, and SCh on the pedicel. The proximal flagellar segments possess SCoI (5–13 μm), SCoII (2.5–4 μm pore), SSt (2.5–3 μm pore), and FO (1–2 μm pore) with a density greater than those on more distal flagellar segments (Figure 2E–H, Figure 3A and Figure 4A–D). A single SCaI (3.2 μm dome in 5 μm depression) was found on the distal portion of the second flagellar segment in *D. apache* (Figure 3B). A single SCh was found near the distal end of the first flagellar segment in *D. eugraphica* and on the lateral surface of the fourth flagellar segment in *D. swalei swalei*. A paired SCoII and FO were found in *D. eugraphica* and *D. semicincta*. SCoI and FO were found near the proximal end of the second flagellar segment in all species, with SCoII also found in *D. canescens*. There are no sensilla on the distal flagellar segments.

#### 3.2.2. Antennal Sensilla in Species of *Beameria* Davis, 1934

All three known species of the genus, *Beameria ansercollis* Sanborn & Heath, 2011, *B. venosa* (Uhler, 1888), and *B. wheeleri* Davis, 1934, were included in the study.

**Figure 5:**
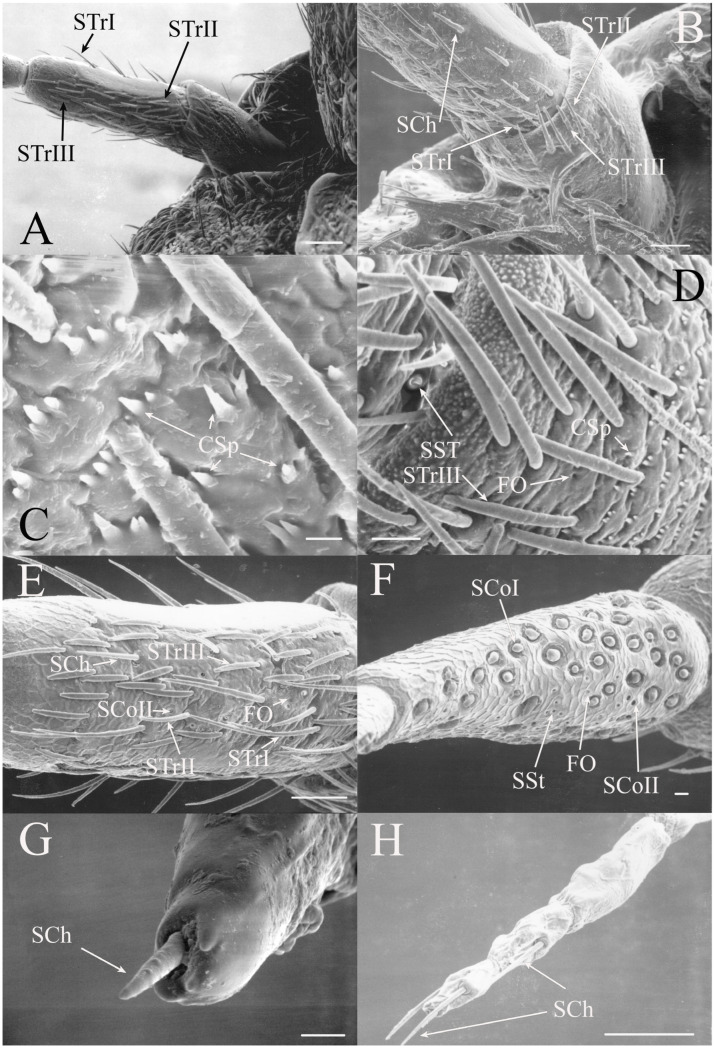
Antennal segments of *Beameria* species. (**A**) Scape and pedicel of *B. venosa*. (**B**) Distal scape and proximal pedicel of *B. wheeleri*. (**C**) Cuticular spines on *B. venosa* scape. (**D**) SSt near distal junction of scape in *B. venosa*. (**E**) Pedicel of *B. venosa*. (**F**) First flagellar segment of *B. venosa*. (**G**) Terminal flagellum of *B. ansercollis*. (**H**) Terminal flagellar segments of *B. wheeleri*. Scale bars: (**A**) = 100 μm, (**B**,**E**,**H**) = 50 μm, (**C**,**G**) = 5 μm, (**D**,**F**) = 10 μm.

**Figure 6:**
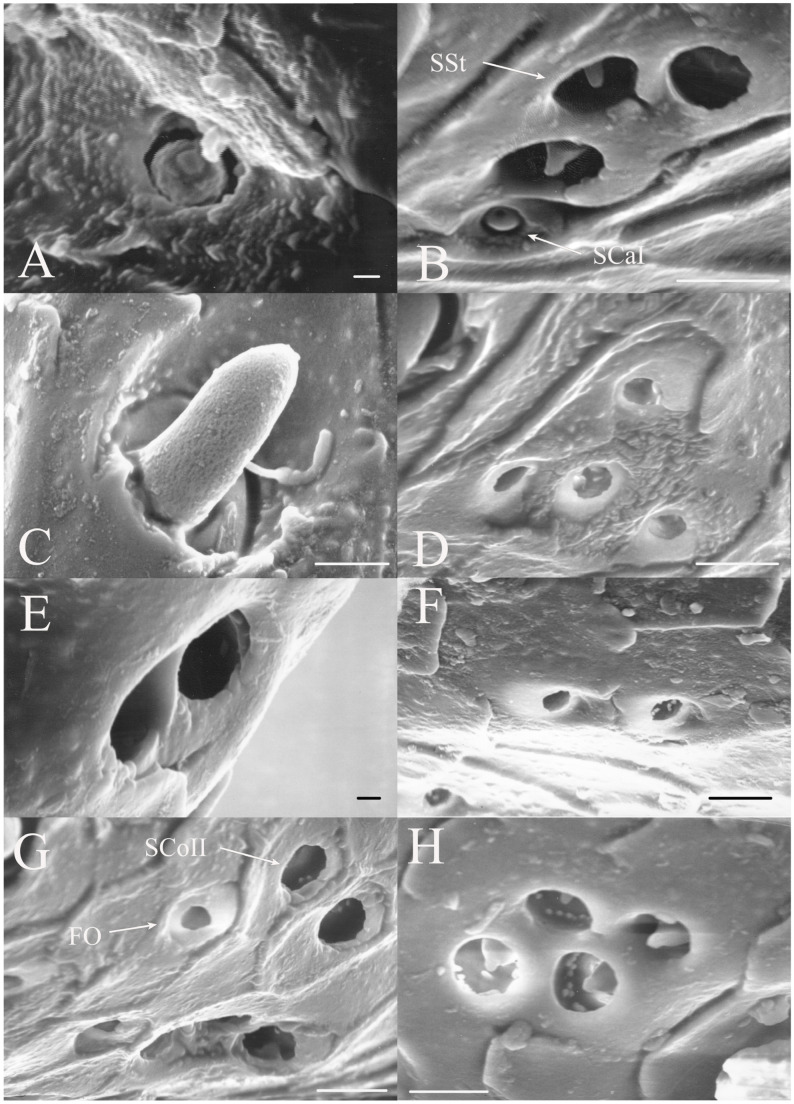
Magnified sensilla of *Beameria* species. (**A**) SCaI on the first flagellar segment of *B. ansercollis*. (**B**) SCaI and group of three SSt on first flagellar segment of *B. wheeleri*. (**C**) SCoI on the first flagellar segment of *B. venosa*. Note the multi-porous surface. (**D**) Group of four SCoII on the first flagellar segment of *B. venosa*. (**E**) Pair of SCoII on the first flagellar segment of *B. venosa*. (**F**) FO on the first flagellar segment of *B. wheeleri*. (**G**) Grouping of SCoII and FO on the first flagellar segment of *B. venosa*. (**H**) Grouping of four SSt on the first flagellar segment of *B. wheeleri*. Scale bars: (**A**,**E**) = 1 μm, (**B**–**D**,**F**–**H**) = 5 μm.

**Figure 7:**
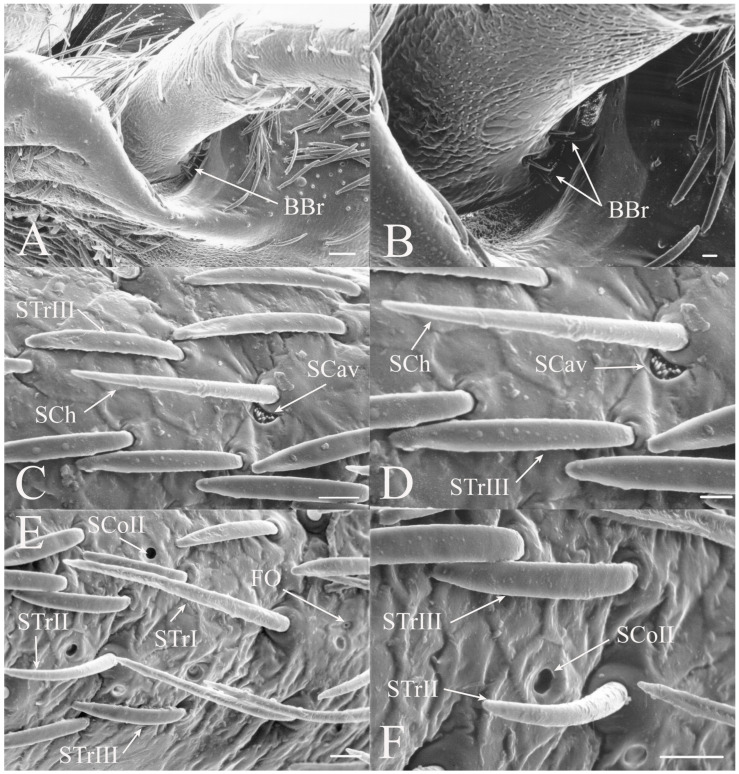
Magnified sensilla of *Beameria* species. (**A**) Böhm’s bristles of *B. venosa*. (**B**) Magnified Böhm’s bristles of *B. venosa*. (**C**) SCav on the first pedicel of *B. venosa*. (**D**) Magnified SCav on the first pedicel of *B. venosa*. (**E**) Sensilla on the pedicel of *B. venosa*. (**F**) Magnified sensilla on the pedicel of *B. venosa*. Scale bars: (**A**) = 50 μm, (**B**,**C**,**E**,**F**) = 10 μm, (**D**) = 5 μm.

All seven types of sensilla are present in the genus: STr, SCh, SCo, SSt, FO, SCaI, and SCav (within 8 μm long and 4 μm wide openings) (Figure 5, Figure 6 and Figure 7). STrI (53–102 μm) and STrII (46–57 μm) are found on the dorsal and ventral surfaces of the pedicel and distal scape, with the greatest density on the ventral side. STrIII (39–52 μm) are also found and are unique to the genus (Figure 5A,B,E). SCh (11–55 μm) are found on the scape, pedicel, and distal flagellar segments of *B. wheeleri*. There is a single SCh near the distal end of the second flagellar segment in *B. wheeleri*, a single SCh on the terminal flagellar segment in *B. ansercollis* (Figure 5G), a single SCh on the penultimate flagellar segment, and a pair of SCh on the terminal flagellar segment of *B. wheeleri* (Figure 5H). SSt (2–4 μm pore) are found on the pedicel and first flagellar segment, with a single SSt found near the distal junction of the pedicel in *B. venosa* (Figure 5D). SSt are found in small groups on the first flagellar segment (Figure 6H). SCoI (8–15 μm) (Figure 6C) and SCoII (2.5–5 μm pore) (Figure 6D,E) are found in their greatest density on the ventral side of the first flagellar segment, with a central gap in their distribution (Figure 5F). SCoII are found primarily near the proximal and distal ends of this central gap (Figure 6E) but were also found distributed on the ventral pedicel. The second flagellar segment also has a few SCoI and SCoII. FO (1–2 μm pore) are found on the first flagellar segment, primarily within this gap of SCoI and SCoII sensilla (Figure 6F,G), but were also seen on the scape and pedicel of *B. venosa* (Figure 7E,F). An SCaI (1.5–2.8 μm dome in a 2.25–3.5 μm depression) was found on the first flagellar segment in *B. ansercollis* (Figure 6A) and *B. wheeleri*. A group of three SSt and one SCaI was found on the first flagellar segment of *B. wheeleri* (Figure 6B). SCav were found at the base of an SCh on the pedicel of *Beameria venosa* (Figure 7C,D). A grouping of about 12 pegs was found in a single crescent-moon-shaped cuticular depression at the base of an SCh. The opening on the antennal surface is 8 μm long and 4 μm wide at its widest point. There is a slightly raised rim on the outer curvature of the depression.

There are two additional structures that were observed while examining the *Beameria* species. Böhm’s bristles (about 22 μm in length) were visible between the scape and the antennal socket of the medial gena ventral to the supra-antennal plate (Figure 7A,B). Cuticular spines from 1 to 5 μm in length are found on the distal portion of the scape in species of *Beameria* (Figure 5C). The number of cuticular spines is significantly reduced in *B. wheeleri* when compared to the other two species. These spines are external modifications of the cuticle and do not appear to have any sensory function.

#### 3.2.3. Antennal Sensilla in Species of *Pacarina* Distant, 1905

A single species, *Pacarina puella* Davis, 1923, was studied.

**Figure 8:**
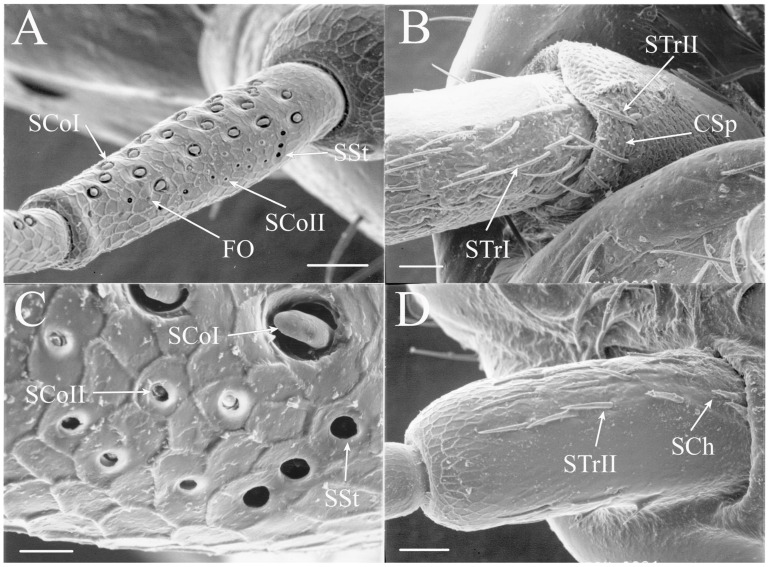
Antennal segments of *Pacarina puella*. (**A**) First flagellar segment. Note the proximal row of SSt. (**B**) Scape and pedicel. Note the cuticular spines on the distal scape. (**C**) Magnified sensilla of the first flagellar segment. (**D**) Magnified pedicel. Scale bars: (**A**,**B**,**D**) = 50 μm, (**C**) = 10 μm.

The antennae of *Pacarina* have reduced sensilla numbers compared to the other Fidicinini species examined (Figure 8). The scape has a few STrII on the distal ventral surface, the pedicel has STrI (66–79 μm), STrII (46–65 μm), and SCh (36–42 μm) primarily on the ventral side with a few sensilla on the dorsal surface (Figure 8B,D). The proximal flagellar segment (Figure 8A,C) has SCoI (8–11.5 μm) along its length, with SCoII (2.75–3 μm pore) and FO (1–2 μm pore) on the ventroanterior surface. There is a row of three SSt (3.5–5 μm pore) proximal to the SCoII and FO (Figure 8A). The second flagellar segment has a few SCoI proximally with SCoII and FO ventroanteriorly and distally. The distal flagellar segments lack sensilla. Finally, cuticular spines (1–4 μm) (Figure 8B), like those found in species of *Diceroprocta* and *Beameria*, were found on the distal portion of the scape in *Pacarina*.

#### 3.2.4. Antennal Sensilla in Species of *Neotibicen* Hill & Moulds, 2015

Two species, *Neotibicen pruinosus pruinosus* (Say, 1825) and *N. superbus* (Fitch, 1855), were included in the analysis.

**Figure 9:**
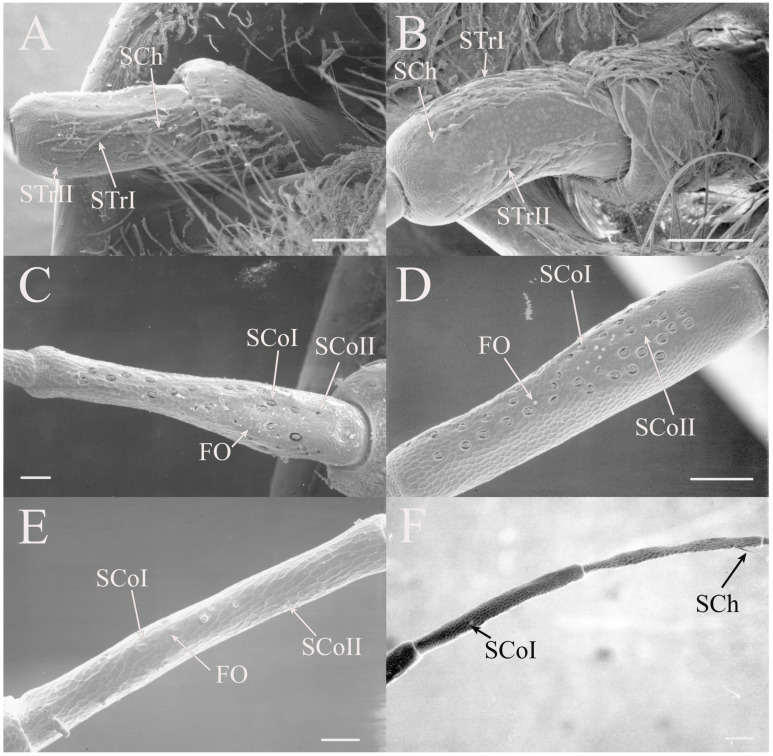
Antennal segments of *Neotibicen* species. (**A**) Scape and pedicel of *N. pruinosus pruinosus*. (**B**) Scape and pedicel of *N. superbus*. (**C**) First flagellar segment of *N. pruinosus pruinosis*. (**D**) First flagellar segment of *N. superbus*. (**E**) Second flagellar segment of *N. pruinosus pruinosis*. (**F**) Second and third flagellar segments of *N. superbus* illustrating distal SCh. Scale bars: (**A**,**B**) = 200 μm, (**C**,**E**) = 50 μm, (**D**,**F**) = 100 μm.

**Figure 10:**
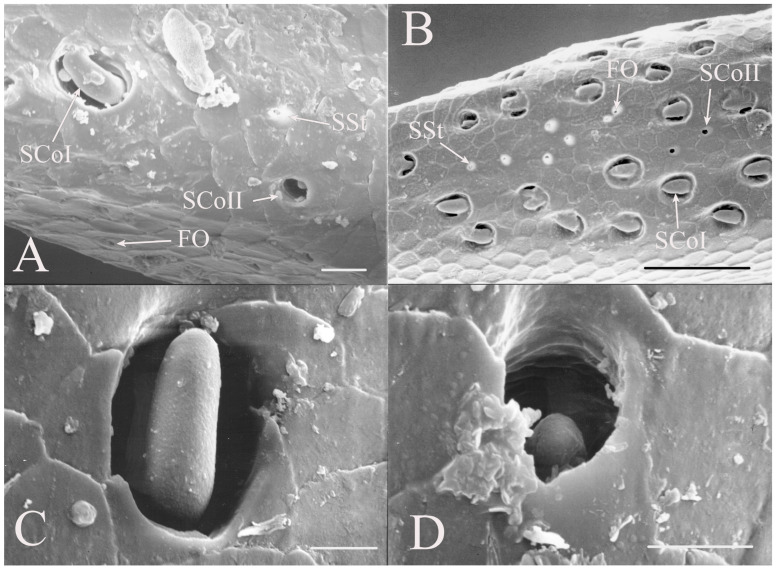
Magnified sensilla of *Neotibicen* species. (**A**) SCoI, SCoII, SSt, and FO on the first flagellar segment of *N. pruinosus pruinosus*. (**B**) SCoI, SCoII, and SSt on first flagellar segment of *N. superbus*. (**C**) SCoI on the first flagellar segment of *N. pruinosus pruinosus*. Note the multi-porous surface. (**D**) SCoII on the first flagellar segment of *N. pruinosus pruinosus* showing sensillum tip within deep depression. Scale bars: (**A**) = 10 μm, (**B**) = 50 μm, (**C**,**D**) = 5 μm.

The antennae of the two species of *Neotibicen* examined have a similar distribution of sensilla (Figure 9 and Figure 10). The pedicel and distal scape possess STrI (130–210 μm), STrII (69–125 μm), and SCh (45–88 μm) on the dorsal surface and in greater density on the ventral surface (Figure 9A,B). The first and second flagellar segments possess SCoI (10–16 μm), SCoII (2.5–5.5 μm pore), SSt (2–2.5 μm pore), and FO (1–1.5 μm pore), with the greatest density on the proximal portion of the first flagellar segment (Figure 9C,D and Figure 10A–D). The proximal half of the second flagellar segment has a few SCoI (Figure 9E,F). There is a single SCh near the distal end of the third flagellar segment in both species and a terminal SCh on the distal flagellar segment in *N. superbus* (Figure 9F) but not in *N. pruinosus pruinosus*.

#### 3.2.5. Antennal Sensilla in Species of *Hadoa* Moulds, 2015

Two species, *Hadoa duryi* (Davis, 1917) and *H. inaudita* (Davis, 1917), were investigated here.

**Figure 11:**
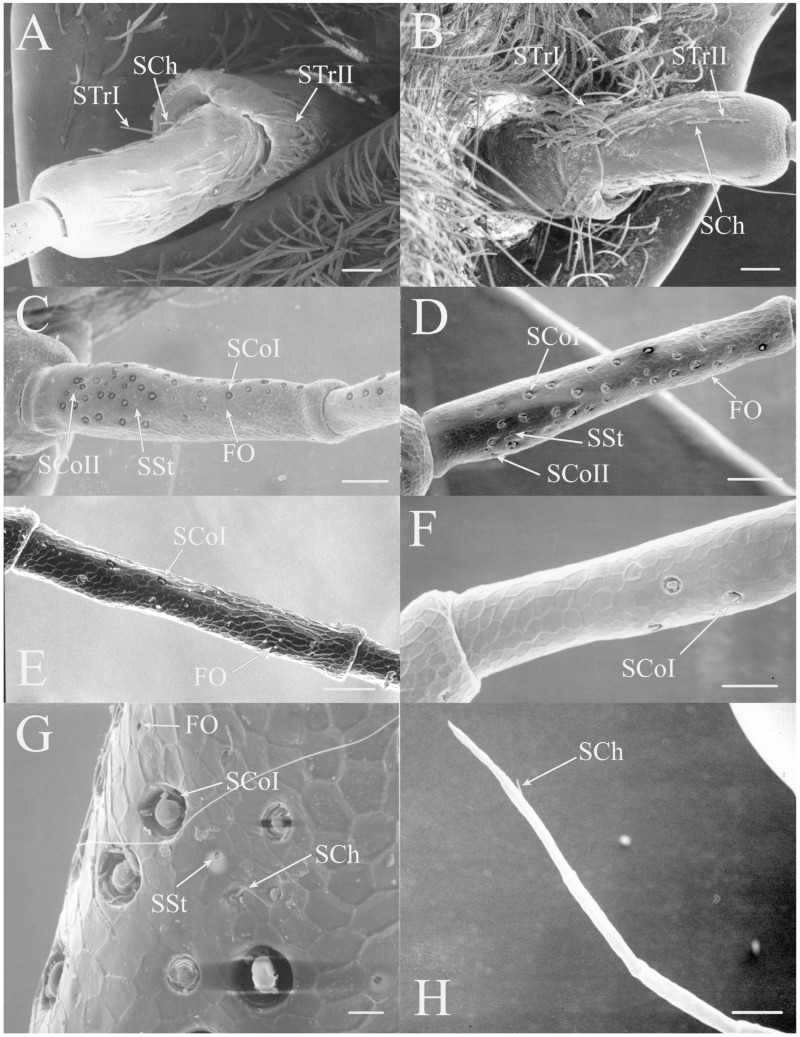
Antennal segments of *Hadoa* species. (**A**) Scape and pedicel of *H. duryi*. (**B**) Scape and pedicel of *H. inauditus*. (**C**) First flagellar segment of *H. duryi*. (**D**) First flagellar segment of *H. inauditus*. (**E**) Second flagellar segment of *H. duryi*. (**F**) Proximal second flagellar segment of *H. inauditus*. (**G**) First flagellar segment sensilla of *H. duryi*. (**H**) Flagellar segments 2–5 of *H. inauditus*. Scale bars: (**A**–**E**) = 100 μm, (**F**) = 50 μm, (**G**) = 10 μm, (**H**) = 200 μm.

The ventrodistal scape and the dorsal and ventral pedicel possess STrI (115–147 μm), STrII (68–78 μm), and SCh (47–65 μm) (Figure 11A,B). The first flagellar segment has SCoI (5–13 μm pore) proximoventrally and distolaterally, along with SCoII (4.5–5 μm), SSt (2–3.5 μm pore), and FO (1–3 μm pore), with small SCh (about 5.5 μm) in *H. duryi* (Figure 11C,D,G). The proximal second flagellar segment has a few SCoI and FO (Figure 11E,F). The third flagellar segment in *H. duryi* has five SCoI proximally. The final distal sensillum is a single SCh near the distal end of flagellar segment 4 in *H. inauditus* (Figure 11H). There are no terminal SCh in the specimens examined.

#### 3.2.6. Antennal Sensilla in Species of *Cacama* Distant, 1904

A single species, *Cacama collinaplaga* Sanborn & Heath, 2011, was studied.

**Figure 12:**
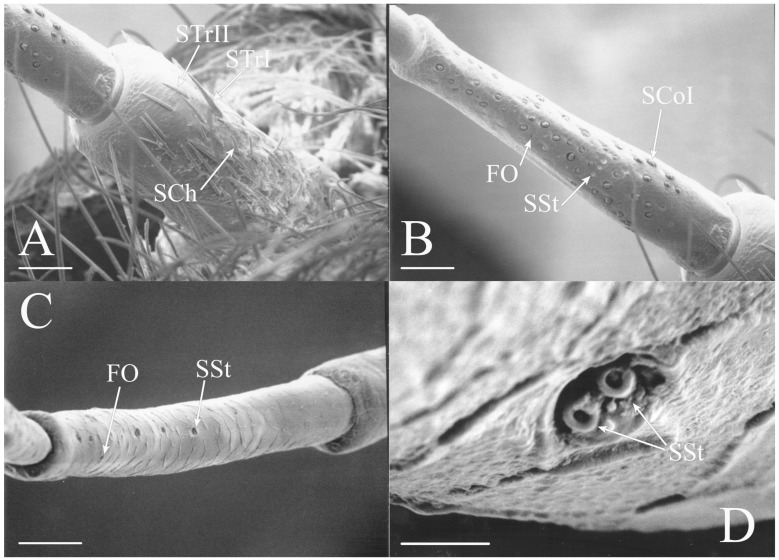
Antennal segments of *Cacama collinoplaga*. (**A**) Pedicel. (**B**) Sensilla on the first flagellar segment. (**C**) Second flagellar segment. (**D**) Magnified pair of SSt on second flagellar segment. Scale bars: (**A**,**B**) = 100 μm, (**C**) = 50 μm, (**D**) = 5 μm.

The scape and pedicel have STrI (83–110 μm), STrII (60–70 μm), and SCh (38–50 μm), with the density greatest on the ventral side (Figure 12A). The dorsal pedicel primarily has SCh receptors. The first flagellar segment has SCoI (6–9 μm) along its length, SSt (1–3 μm pore), and FO (1–2 μm pore) (Figure 12B). The second flagellar segment possesses a limited number of SCoI along its length and medial and distal FO (Figure 12C). A unique form of SSt was found on the second and third flagellar segments (Figure 12D). There is a pair of SSt receptors in an angled cuticular depression with a raised rim surrounding the pore. There are no SCh on the terminal flagellar segments.

#### 3.2.7. Antennal Sensilla in Species of *Neocicada* Kato, 1932

A single species, *Neocicada chisos* (Davis, 1916), was examined.

**Figure 13:**
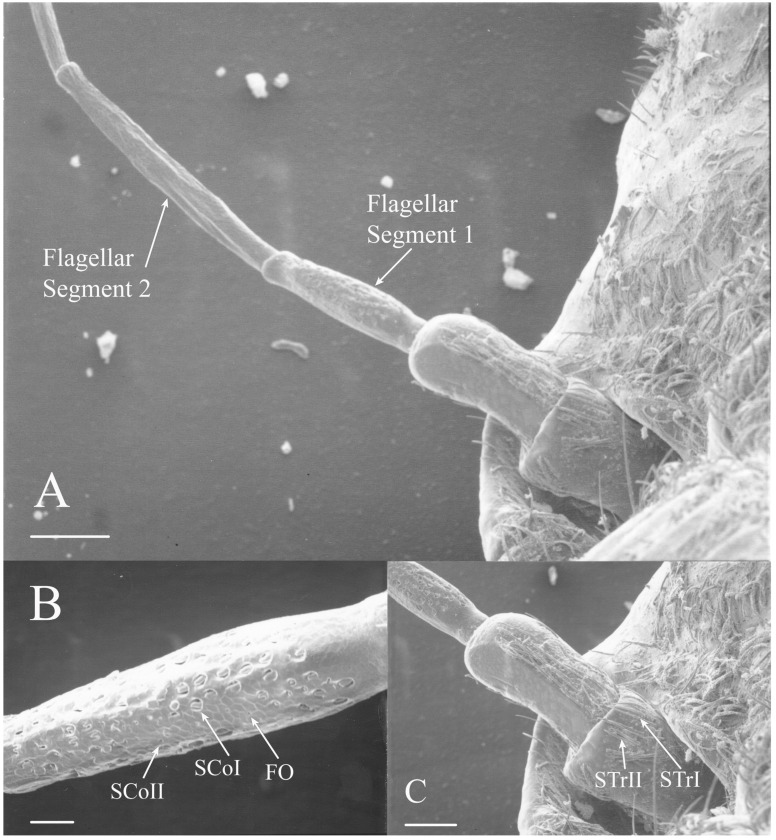
Antennal segments of *Neocicada chisos*. (**A**) Proximal portion of antenna from the scape to the base of flagellar segment 3. Note the second flagellar segment is longer than the first flagellar segment, a character unique to the genus. (**B**) Sensilla on the first flagellar segment. (**C**) Scape and pedicel. Scale bars: (**A**) = 300 μm, (**B**) = 50 μm, (**C**) = 200 μm.

The dorsal and ventral pedicel and distal scape have STrI (154–210 μm), STrII (100–125 μm), and a limited number of SCh (64–76 μm) (Figure 13A,C). The first flagellar segment has SCoI (6–13 μm) along its length, a few SCoII with wide pores proximally (2.8–6.4 μm pore), a few SSt (2.5–3.5 μm pore), and a few FO (1.8–2 μm pore) (Figure 13B). The distal flagellar segments lack sensilla.

The most unique feature of the *N. chisos* antenna is the proportionately longer second flagellar segment (Figure 13A). In other species examined, the second flagellar segment is shorter than the first flagellar segment. In *N. chisos*, the second flagellar segment is about 1.75 times longer than the first flagellar segment. This is particularly odd since there are no sensilla associated with the second flagellar segment.

#### 3.2.8. Antennal Sensilla in Species of *Magicicada* Davis, 1925

Four species, *Magicicada cassinii* (Fisher, 1852), *M. septendecim* (Linnaeus, 1758), *M. septendecula* Alexander & Moore, 1962, and *M. neotredecim* Marshall & Cooley, 2000, were included in this analysis. The antennae of *M. cassinii* were investigated by Klein et al. [3], who also determined the innervation patterns of some sensilla, which helps in the process of hypothesizing the function of the various types of sensilla.

**Figure 14:**
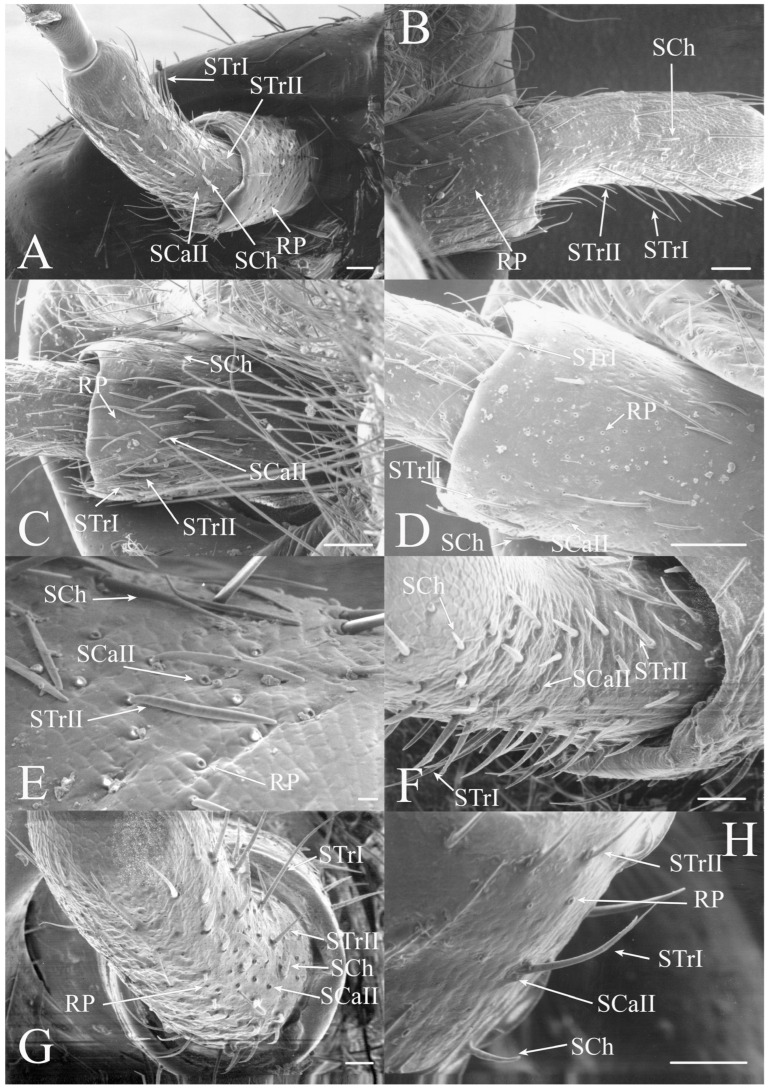
Scape and pedicel of *Magicicada* species. (**A**) Scape and pedicel of *M. septendecim*. (**B**) Scape and pedicel of *M. neotredecim*. (**C**) Ventral scape of *M. cassinii*. (**D**) Lateral scape of *M. cassinii*. (**E**) Magnified scape of *M. neotredecim*. (**F**) Pedicel of *M. septendecula*. (**G**) Pedicel of *M. septendecim*. (**H**) Magnified pedicel of *M. septendecula*. Scale bars: (**A**–**D**) = 100 μm, (**E**) = 10 μm, (**F**–**H**) = 50 μm.

**Figure 15:**
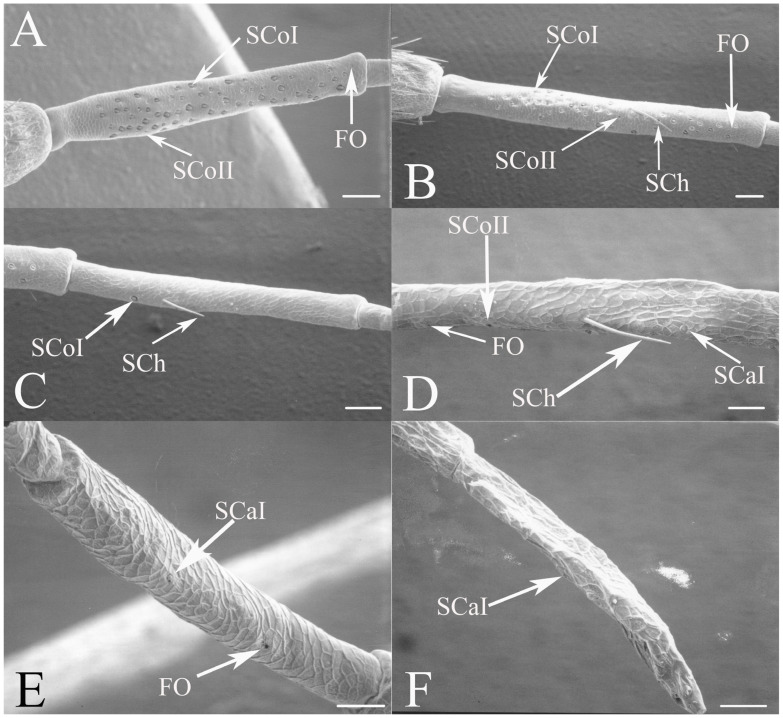
Flagellar segments of *Magicicada* species. (**A**) First flagellar segment of *M. cassinii*. (**B**) First flagellar segment of *M. neotredecim*. (**C**) Second flagellar segment of *M. neotredecim*. (**D**) Third flagellar segment of *M. neotredecim*. (**E**) Fourth flagellar segment of *M. cassinii*. (**F**) Fifth flagellar segment of *M. neotredecim*. Scale bars: (**A**–**C**) = 100 μm, (**D**–**F**) = 50 μm.

**Figure 16:**
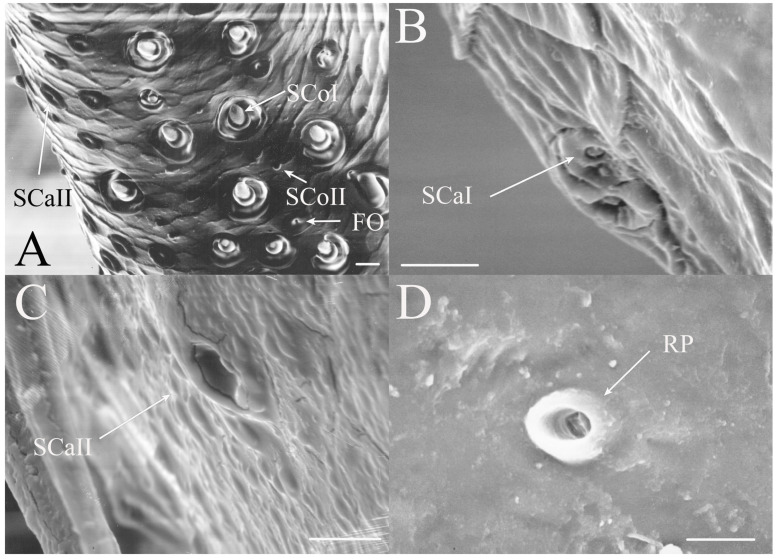
Magnified sensilla of *Magicicada* species. (**A**) SCoI, SCoII, SCaII, and FO on the first flagellar segment of *M. septendecim*. Note the smaller SCoI identified as medium sensillum by Klein et al. [3]. (**B**) SCaI on fifth flagellar segment of *M. neotredecim*. (**C**) SCaII on the first flagellar segment of *M. cassinii*. (**D**) Rimmed pore on the pedicel of *M. cassinii*. Scale bars: (**A**) = 10 μm, (**B**–**D**) = 5 μm.

Six types of sensilla were identified on the various antennal segments. Trichoid sensilla (STrI 215–340 μm, STrII 55–85 μm) and sensilla chaetica (35–120 μm) were found on the dorsal and ventral scape and pedicel (Figure 14A–H). Unlike most species examined, the hair-like sensilla are also found on the lateral surfaces of the scape and pedicel, with fewer lateral sensilla found in *M. septendecim* and *M. neotredecim*. Klein et al. [3] found a single sensillum chaeticum on the second flagellar segment and a pair of sensilla chaetica on the terminal flagellar segment in *M. cassinii*, with a single sensillum chaeticum found on each on the proximal three flagellar segments of *M. neotredecim* (Figure 15B–D). The flagellar segments (Figure 15A–F and Figure 16A) have the sensilla concentrated on the ventrolateral surface. SCoI (9–14 μm) is the most numerous type of sensillum, but SCoII (4–6.5 μm pore) and FO (1–3 μm pore) are also found on the first and proximal half of the second flagellar segment. Paired SCoI within a single cuticular depression were found in one or two locations on the first flagellar segment. Paired or triplet SCoI were also found by Klein et al. [3]. Based on the variability in number, the variability of position, and the shape of the pores, it appears that the pores may have fused during development, producing the doublet or triplet sensilla within the individual depressions, in contrast to what is described in *Tibicinoides*, *Okanagana*, and *Platypedia*, below. SSt were found on the second flagellar segment of *M. cassinii*. FO were found on the third flagellar segment of *M. cassinii* and *M. neotredecim*. SCaI (a 1–20 μm dome in a 1.5–2.5 μm depression) were reported on the scape, pedicel, and distal first flagellar segment in *M. cassinii* [3] and were found on flagellar segments 3–5. SCaI were also found in *M. neotrecedim* on the scape and as a structure of paired SCaI on the terminal flagellar segment (Figure 16B). A sensillum subclass unique to *Magicicada* is SCaII (8–12 μm in diameter with a 2.5–6 μm pore) (Figure 16C), generally found on the scape and pedicel, but found in greatest number on the first flagellar segment of *M. septendecim* (Figure 16A). The classical form is found on the scape and pedicel of *M. cassinii* [3]. The SCaII found here are distributed among the rimmed pores (5–6.5 μm diamater) (Figure 16D) found on the scape and pedicel and on the first flagellar segment.

#### 3.2.9. Antennal Sensilla in Species of *Tibicinoides* Distant, 1914

Three species of the genus, *Tibicinoides hesperia* (Uhler, 1872), *T. rubrovenosa* (Davis, 1915), and *T. utahensis* (Davis, 1919), were included in this study. These species were classified in the genus *Okanagana* until recently [13].

**Figure 17:**
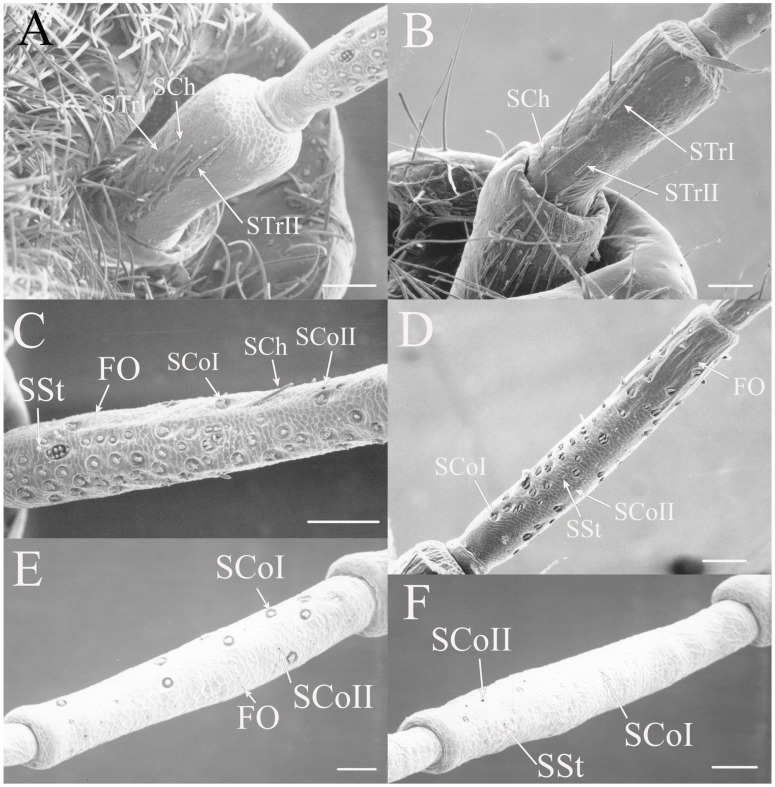
Antennal segments of *Tibicinoides* species. (**A**) Distal scape and pedicel of *T. hesperia*. (**B**) Scape and pedicel of *T. rubrovenosa*. (**C**) First flagellar segment of *T. hesperia*. (**D**) First flagellar segment of *T. rubrovenosa*. (**E**) Second flagellar segment of *T. hesperia*. (**F**) Second flagellar segment of *T. rubrovenosa*. Scale bars: (**A**–**D**) = 100 μm, (**E**,**F**) = 50 μm.

**Figure 18:**
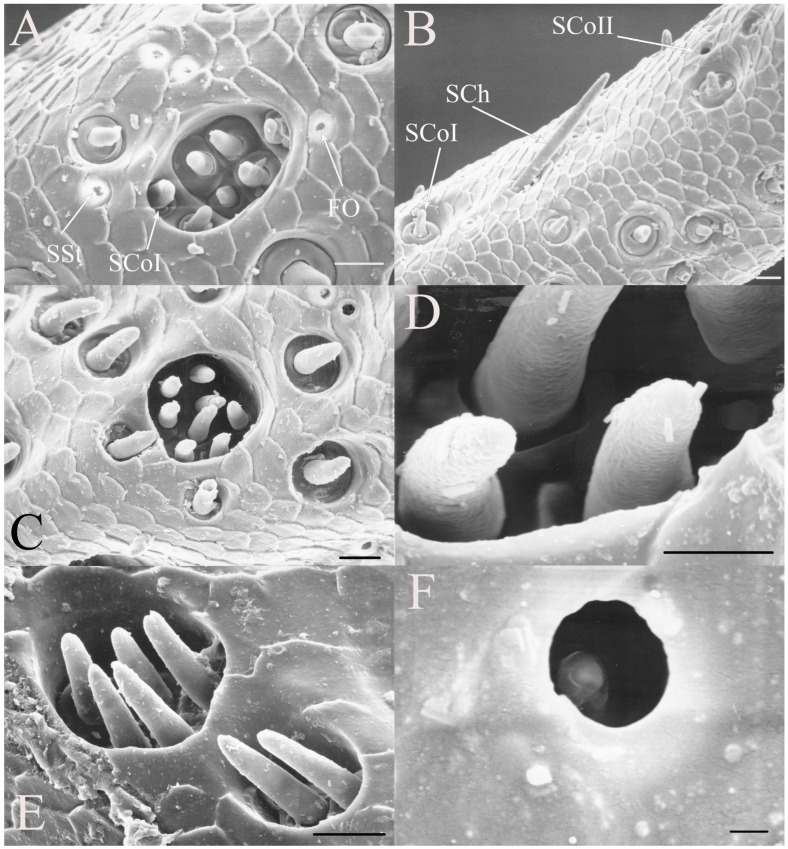
Antennal sensilla of *Tibicinoides* species. (**A**) Grouping of SCoI on the first flagellar segment of *T. hesperia*. (**B**) Distal end of first flagellar segment of *T. hesperius*. (**C**) Grouping of SCoI on the first flagellar segment of *T. rubrovenosa*. (**D**) Magnified grouping of SCoI on the first flagellar segment of *T. rubrovenosa*. (**E**) Smaller grouping of SCoI on the first flagellar segment of *T. rubrovenosa*. (**F**) Magnified SCoII on the first flagellar segment of *T. rubrovenosa*. Scale bars: (**A**–**C**,**E**) = 10 μm, (**D**) = 5 μm, (**F**) = 1 μm.

The antennae of *Tibicinoides* species possess a scape and pedicel with STrI (85–164 μm), STrII (72–86 μm), and SCh (28–67 μm) on the dorsal and ventral surfaces (Figure 17A,B). The first flagellar segment contains SCoI (6.5–18 μm), SCoII (2.5–4 μm pore), SSt (1.5–2 μm pore), and FO (1–1.5 μm pore), with a single distal SCh in *T. hesperia* (Figure 17C and Figure 18A,B). There is a single grouping of SCoI (Figure 17C,D and Figure 18A,C–E) in the proximocentral region of the first flagellar segment that is unique to *Tibicinoides* and *Okanagana*. This grouping of sensilla is found within a single cuticular depression and contains between four and nine individual sensilla. The second flagellar segment (Figure 17C,F) possesses a few SCoI, SCoII, and FO sensilla, with *T. hesperia* having SCoI along most of the segment length (Figure 17E). There are a couple distal SCoI, SCoII, and SSt on the third flagellar segment well. There are two sizes of SCoI on the flagellar segment. Another divergence in the structure of the SCoI in *Tibicinoides* is the large basal region surrounding the sensillum. Sensilla styloconica are seen on the first and third flagellar segments of *Tibicinoides* species. Foramina olfactoria are found on the proximal three flagellar segments, with doublets found on the distal third flagellar segment of *T. rubrovenosa* and *T. utahensis*.

The grouping of SCoI found on the first antennal segment of *Tibicinoides* species is composed of multi-porous coeloconic sensilla, with each sensillum protruding from an individual hole in the chitin. This suggests they are derived from individual trichogen and tormogen cells and not produced by incomplete mitosis. This structure is also found in most species of *Okanagana*, although the number of sensilla within the grouping is fewer in *Okanagana*.

#### 3.2.10. Antennal Sensilla in Species of *Okanagana* Distant, 1905

Four species, *Okanagana fumipennis* Davis, 1932, *O. magnifica* Davis, 1919, *O. mariposa mariposa* Davis, 1915, and *O. synodica atrata* Dmitriev, 2020, were included in the analysis.

**Figure 19:**
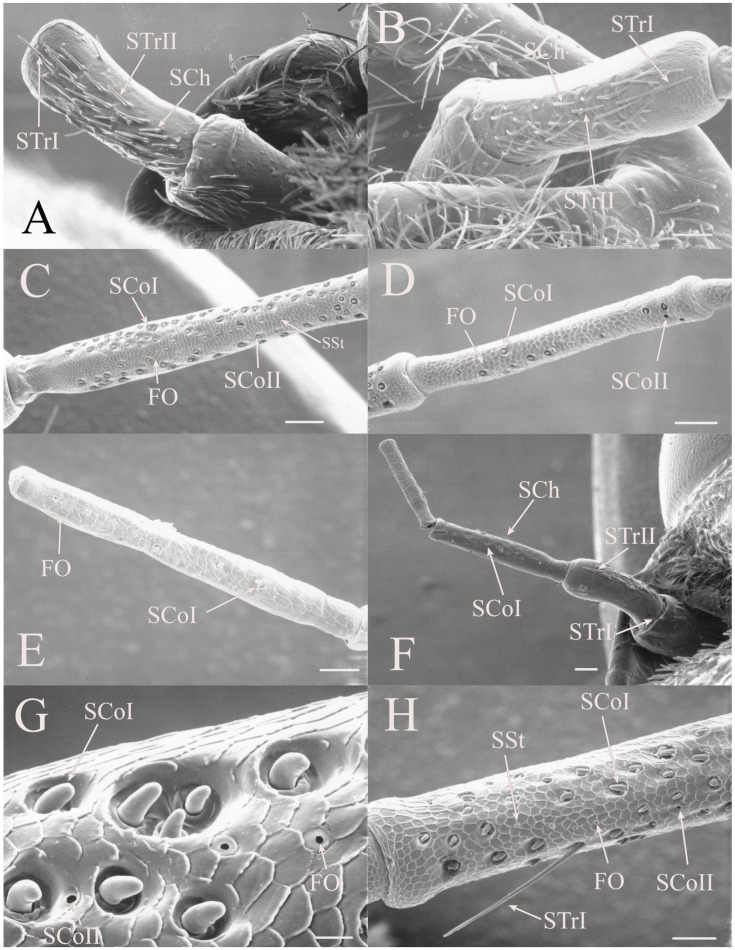
Antennal segments of *Okanagana* species. (**A**) Scape and pedicel of *O. fumipennis*. (**B**) Scape and pedicel of *O. mariposa mariposa*. (**C**) First flagellar segment of *O. mariposa mariposa*. (**D**) Second flagellar segment of *O. mariposa mariposa*. (**E**) Fourth flagellar segment of *O. mariposa mariposa*. (**F**) Scape, pedicel, and first flagellar segment of *O. synodica atrata*. Note the reduction in sensilla on all segments. (**G**) Sensilla on first flagellar segment of *O. mariposa mariposa*, including a grouping of SCoI. (**H**) Distal end of first flagellar segment of *O. mariposa mariposa*. Scale bars: (**A**–**D**,**F**) = 100 μm, (**E**,**H**) = 50 μm, (**G**) = 10 μm.

Like *Tibicinoides*, the antennae of all species of *Okanagana* posses a scape and pedicel possessing primarily STrI (164–190 μm), STrII (86–112 μm), and SCh (54–70 μm) (Figure 19A,B,F). The first flagellar segment has the greatest number of sensilla, with SCoI (9–20 μm), SCoII (2.5–4 μm pore), SSt (2.5–3 μm pore), and FO (1.5–2 μm pore) present, with a single STrI near the distal end of the segment in *O. mariposa mariposa* (Figure 19C,G,H). There is a grouping of SCoI within a deep pore with three or four sensilla present, similar to the grouping found in *Tibicinoides* species. The second flagellar segment (Figure 19D) has SCoI, SCoII, and FO, with one doublet of SCoI in *O. magnifica*. SCoI, SCoII, SSt, and FO were found on the third flagellar segment and SCoI and FO were found on the fourth flagellar segment of *O. mariposa mariposa* (Figure 19E). The antennae of *O. synodica atrata* are unusual in that they contain only a few trichoid sensilla and coeloconic sensilla, with two SCh found on the first flagellar segment (Figure 19F).

The grouping of coeloconic sensilla found in *Tibicinoides* is also found in most species of *Okanagana* that were studied, but fewer sensilla (three or four vs. up to nine) were included in the grouping. The exception is the antenna of *O. synodica atrata*, where the grouping is absent. This may be another example of simplification of antennal structure due to the grassland inhabited by the species [8], as seen with other sensilla in the species.

#### 3.2.11. Antennal Sensilla in Species of *Okanagodes* Davis, 1919

Both species of the genus, *Okanagodes gracilis gracilis* Davis, 1919 and *O. terlingua* Davis, 1932, were examined here.

**Figure 20:**
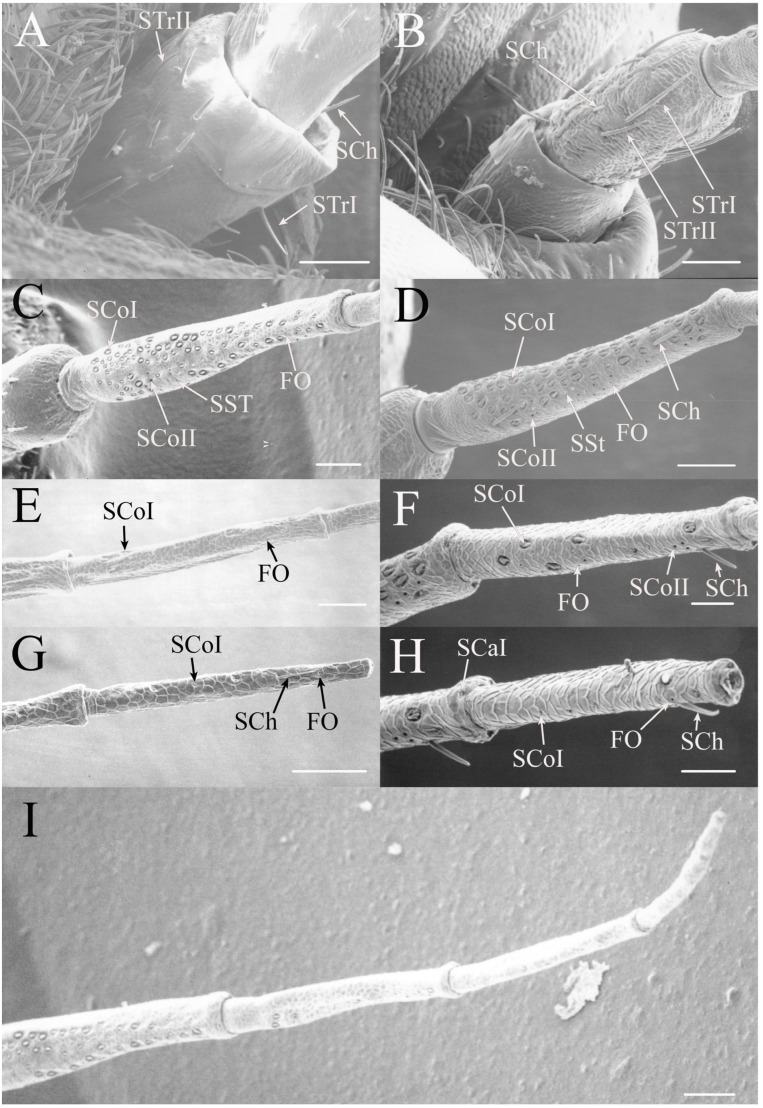
Antennal segments of *Okanagodes* species. (**A**) Scape of *O. gracilis gracilis*. (**B**) Scape and pedicel of *O. terlingua*. (**C**) First flagellar segment of *O. gracilis gracilis*. (**D**) First flagellar segment of *O. terlingua*. Note the SCh not found in *O. gracilis gracilis*. (**E**) Second flagellar segment of *O. gracilis gracilis*. (**F**) Second flagellar segment of *O. terlingua*. (**G**) Third flagellar segment of *O. gracilis gracilis*. (**H**) Third flagellar segment of *O. terlingua*. (**I**) Terminal flagellar segments of *O. gracilis gracilis*. Scale bars: (**A**–**E**,**G**,**I**) = 100 μm, (**F**,**H**) = 50 μm.

**Figure 21:**
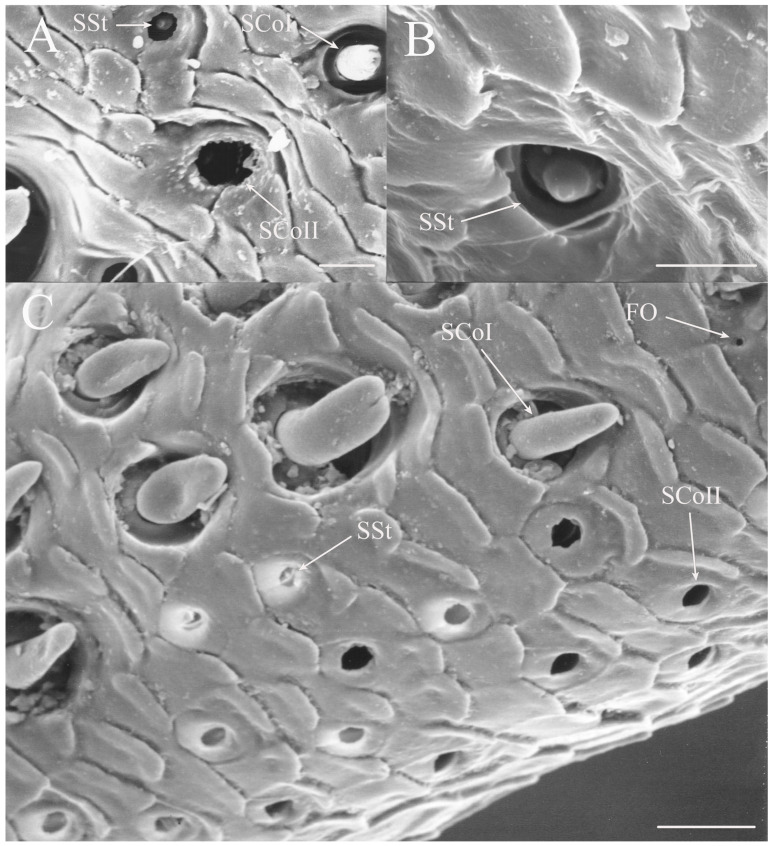
Magnified sensilla of *Okanagodes* species. (**A**) Grouping of SCoI, SCoII, and SSt on the first flagellar segment of *O. gracilis gracilis* illustrating sensilla within depressions. (**B**) SSt of *O. gracilis gracilis* illustrating sensillum. (**C**) Central aggregation of SCoII and SSt within SCoI on the first flagellar segment of *O. terlingua*. Scale bars: (**A**,**B**) = 5 μm, (**C**) = 10 μm.

The number of hair-like sensilla on the scape and pedicel in *Okanagodes* is less than found in most species (Figure 20A,B). The distribution of the STrI (83–137 μm), STrII (49–90 μm), and SCh (22–66 μm) is similar to other cicadas, with the greatest density on the ventral pedicel. In addition, *O. gracilis gracilis* has fewer STrI than *O. terlingua* (Figure 20A,B). The proximal flagellar segment has SCoI (9–12 μm), in two distinct sizes, a few SCoII (2.5–4 μm pore), SSt (2–4.5 μm pore), and FO (1–1.5 μm pore) (Figure 20C,D and Figure 21A–C). The SSt and SCoII are concentrated on the anteroventral side. In addition, *O. terlingua* has one proximal and one distal SCh on the first flagellar segment (Figure 20D). The second flagellar segment (Figure 20E,F) has SCoI and a few FO, with a single distal SCh and distal SCaI (a 3.3 μm dome in a 4 μm depression) found in *O. terlingua* (Figure 20F). The third flagellar segment (Figure 20G,H) has a few of the smaller SCoI and a distal FO, with *O. terlingua* having a pair of SCh near the distal end.

#### 3.2.12. Antennal Sensilla in Species of *Platypedia* Uhler, 1888

A single species, *Platypedia balli* Davis, 1936, was included in this study.

**Figure 22:**
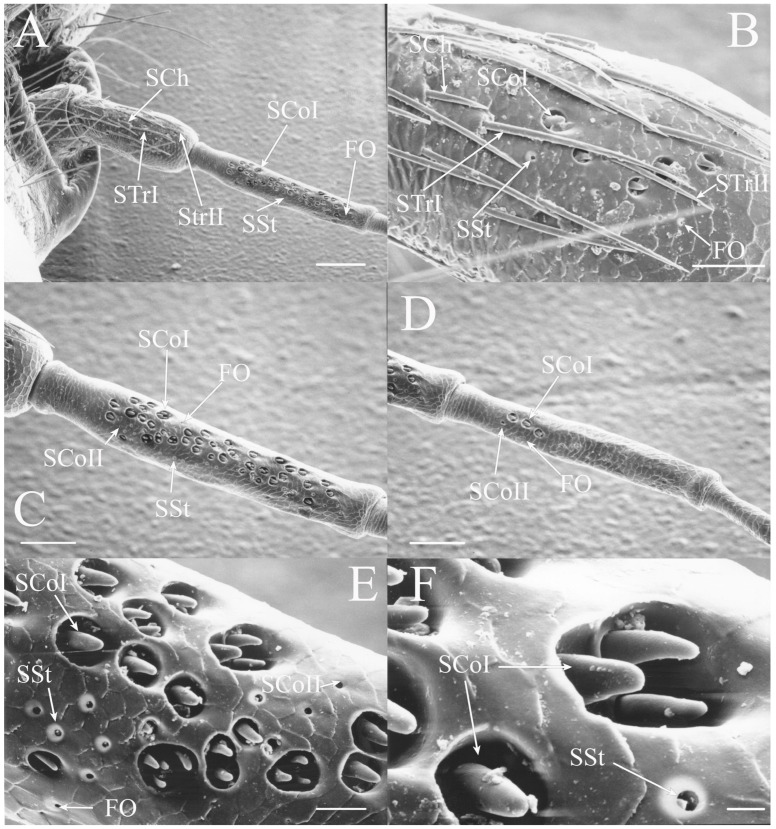
Antennal segments of *Platypedia balli*. (**A**) Proximal portion of antenna from the scape to the base of flagellar segment 2. Note the multiple groups of SCoI on the first flagellar segment, a character unique to the genus. (**B**) Ventral pedicel sensilla. (**C**) Sensilla on the first flagellar segment. (**D**) Sensilla on flagellar segment 2. (**E**) Magnified sensilla on the first flagellar segment. (**F**) Magnified group of SCoI and SSt. Scale bars: (**A**) = 200 μm, (**B**) = 50 μm, (**C**,**D**) = 100 μm, (**E**) = 20 μm, (**F**) = 5 μm.

The antennae of *Platypedia* show several divergent characteristics (Figure 22). The dorsal and ventral distal scape and the dorsal and ventral pedicel have STrI (239–310 μm), STrII (50–168 μm), and SCh (25–87 μm) (Figure 22A,B). The ventral pedicel also has SCoI, SSt, and FO present (Figure 22B). The first flagellar segment has SCoI (9–18 μm), SCoII (2–5 μm pore), SSt (2.5–3.5 μm pore), and a few FO (1–1.5 μm pore) (Figure 22C,E,F). The unique feature is the arrangement of the SCoI on the first flagellar segment. There are seven groups of between two and five sensilla within a single depression reminiscent of the groupings of sensilla found in *Tibicinoides* and *Okanagana*. The second flagellar segment has three proximal SCoI and one proximal FO, with a few SCoII present as well (Figure 22D).

## 4. Discussion

Seven main types of antennal sensilla, sensilla trichodea, sensilla chaetica, sensilla coeloconica, sensilla styloconica, foramen olfactorium, sensilla campaniformia, and sensilla cavitata-peg, along with cuticular spines were found in the North American cicadas. All sensilla types were also found in Asian species [4,5], but there are some unique subtypes in the North American fauna. The sensilla basiconica of Wang et al. [5] are considered to be equivalent to the sensilla chaetica described here based on their bristle-like shape, well-defined socket, and their previous classification as sensilla chaetica in Li and Wei [4].

The basic antennal morphology was consistent in all species studied, with the exception of the second flagellar segment of *Neocicada* being longer (about 1.75×) than the first flagellar segment. Unique receptors or forms were found in a few genera or species. STrIII were only found on the scape and pedicel of *Beameria* species. Grouped SCoI were found centrally located on the first flagellar segment of Tibicininae species. A single grouping was found in species of the genera *Tibicinoides* and *Okanagana* (Tibicinini), while seven groups, composed of two to five sensilla, were found in the species of *Platypedia* (Platypediini) studied. A similar grouping of coeloconic sensilla has been reported in beetles [27]. Half of the species of *Diceroprocta* studied have a grouping of several receptors in a single SCoII on the pedicel. Paired SSt or FO were found on distal flagellar segments of *Cacama* and *Tibicinoides*. The SCaII were only found on the scape, pedicel, and first flagellar segment of *Magicicada* species. The SCaII structure is similar morphologically to campaniform sensilla found in mosquitoes [28], and the structures were found in groups oriented in the same direction, as has been described for other campaniform sensilla [25].

An evolutionary convergence was found in the grass-dwelling species of *Beameria* and *Okanagana synodica atrata*. There is a reduction in the total number of sensilla on the antennae, with the trichoid sensilla found only on the dorsal and ventral surfaces and noticeably absent from the lateral surfaces of the pedicel, and they are found only on the distal scape in all grass-dwelling species. The first flagellar segment also has an absence of SCoI along the medial region of the segment, which only has SCoII, SSt, and FO sensilla. The pattern in *O. synodica atrata* is divergent from other *Okanagana* species studied which live in trees [8] and is convergent with the grass-dwelling species of *Beameria*, which are classified in a different subfamily.

Although external structure can be used to classify the types of receptors present, determining the function requires information on the innervation patterns of the receptors. The number and types of neurons innervating an individual sensillum and a single- or double-wall construction provide significant information on the potential function of the sensillum [22,26]. Obtaining this information requires sectioning the antennae to determine the fine structures and cell types found in the individual sensilla, which has only been performed for one species of cicada [3]. Only electrophysiological techniques can definitively determine the function of any particular sensillum type. The following discussion is based on comparative morphology and functions determined for the various types of sensilla in different insects.

Setaceous antennae are especially effective in deciphering mechanoreceptive information, particularly touch and movement. These functions are crucial for flight stabilization and navigation. The concentration of trichoid sensilla on the scape and pedicel and the sensilla chaetica at the base of the antennae suggest that these receptors are used to influence cicada flight, as has been shown in other insects, e.g., [29,30,31]. The few distal trichoid sensilla found may be used in tactile communication, e.g., as in courtship, or potentially function in olfaction or taste if there are pores associated with the tip. The other types of receptors on the antennae can provide sensitivity to odorants, temperature, and humidity.

Sensilla trichodea and sensilla chaetica are hair-like structures that differ in the potential presence of a terminal pore or pores in the wall of some trichoid sensilla. Both sensilla trichodea and sensilla chaetica are mechanoreceptors, while trichoid sensilla may also function in olfaction or gestation [22]. The walls of sensilla chaetica are too thick to permit the transmission of either olfactory or gustatory molecules [18], but these sensilla have been shown to be important in sensing antennal position and as gravity receptors [19]. Trichoid sensilla were found in all species examined, primarily located on the scape and pedicel. The distribution would support a mechanoreceptive function and their possible use in determining antennal position, or they may act as gravity receptors [19]. The Böhm’s bristles are probably used to determine antennal position.

Sensilla coeloconica were also found in all species examined. The presence of pores on the surface of many of these receptors is characteristic of olfactory receptors [20]. Pores in the sensilla wall have been shown to be the primary olfactory sites in insects allowing odorant molecules to reach the receptor molecules on the neurons [22]. The presence of numerous pores on the surface of the majority of these receptors and the density of these receptors on the first flagellar segment in cicadas suggests that they are the primary olfactory receptors on the antennae. Unpublished preliminary electroantennagrams recorded by the author showed that cicada antennae respond to a number of plant volatiles (the ester isoamyl acetate, the alkane hexane, the alcohol hexanol, and the aldehydes hexanal and trans-2-hexanal), demonstrating that the cicada antennae are sensitive to volatile plant compounds and are probably used in host plant recognition. Klein et al. [3,32] also had limited success in recording electrical responses from individual neurons but showed that the different neuron types are responsive to a variety of chemical compounds (aldehydes, alcohols, acids, esters, ketones, and hydrocarbons). The responsiveness to chemicals found in plants that are not hosts [3,32] may decrease the likelihood of ovipositing in a plant that will have a low chance of success in supporting successful hatching, e.g., [33].

Although the sensilla coeloconica are primarily olfactory receptors, these sensilla can also function in thermoreception and hygroreception [20,22,25,34,35,36]. Sensilla coeloconica can decipher multiple sensory modalities within individual sensilla so that a single sensillum may be responsive to olfaction, temperature, and/or humidity [22]. The innervation patterns in the small coeloconic sensilla in *Magicicada* suggests a combination thermoreceptor and hygroreceptor [3]. The grouped sensilla found on the first flagellar segment of species of *Tibicinoides*, *Okanagana*, and *Platypedia* and possibly the paired sensilla found in distal flagellar segments in *Beameria* and possibly the paired or triplet sensilla found by Klein et al. in *Magicicada* [3] (but not observed in the specimens of the same species examined here) probably function in deciphering more than one sense. The smooth surface of the receptor in some species suggests that it functions in either thermoreception or hygroreception [22], while the corrugated surface in other species suggests an olfactory function [25]. Cicadas have been shown to select specific microclimates during the day [37], so the ability to sense temperature and humidity is supported by their behavior in their natural environment.

The grouping of coeloconic sensilla found in species of *Tibicinoides*, *Okanagana*, and *Platypedia* probably functions in deciphering more than one sensory modality. The multi-porous surface suggests that the sensilla are involved in olfaction but may also function as thermoreceptors and/or hygroreceptors [22]. These appear to be a distinct class of receptors found in these two genera as the pores on the doublet or triplet coeloconic sensilla on the antennae of *Magicicada* appear to be the result of development, since the edges of two or three pores appear to fuse to form a single opening and there is variability in the number and position of these doublets and triplets rather than their always being found in the same location on the antennae as they are in *Tibicinoides* and *Okanagana*, and the number and distribution of these groups in *Platypedia* suggest these are specific developmental outcomes, and these doublets or triplets were not observed in the specimens of *M. cassinii* examined here. In addition, the dendrites insulating the sensory neurons of these doublets or triplets are surrounded by a single dendritic sheath rather than individually, as found in the single receptors [3].

Sensilla styloconica are similar to sensilla coeloconica in their functions. The lack of pores in the surface of the sensillum and their position below the surface of the antennae suggest that they function in thermoreception and/or hygroreception [22,26]. They were found primarily on the first flagellar segment but were also observed on the pedicel and the second and third flagellar segments. The sensilla were also observed in groups of two or three sensilla next to one another in various species.

The foramen olfactorium is a small pore on the surface of the antenna. The function is thought to be olfactory, as stated in the name [5]. There is a possibility that these pores are the openings to deep coeloconic sensilla, as found in *Magicicada* [3], but sectioning of the antenna is required to determine the internal structure and whether they are actually a third form of coeloconic sensillum or have a different deep morphology.

Receptors with the classic sensillum campaniformia (SCaI) morphology were found on the scape, pedicel, and flagellar segments. However, there is an additional subclass found only on the scape, pedicel, and first flagellar segment in species of *Magicicada*. The SCaII can be characterized morphologically as a cup without a small basiconic sensillum at its base. This cicada sensillum is morphologically similar to that found in mosquitoes [31]. Sensilla campaniformia have been described as resembling empty hair sockets [34] and are often found in groups oriented in the same direction so that they function as a unit [25]. Sensilla campaniformia have been shown to function as air pressure receptors on the antennae of multiple insect groups that help to regulate flight [38], but it has also been suggested that they function as hygroreceptors [35]. Klein et al. [3] suggested that the classically designed sensilla were mechanoreceptors with a single sensory cell innervating the dome, which is probably the case for the campaniform sensilla described here.

The sensilla cavitata-peg described by Wang et al. [5] was found only at the base of a single SCh in *Beameria venosa* among the North American taxa. These receptors were hypothesized to be chemoreceptors based on the surface pores found on the pegs [5]. The sensilla cavitata-peg receptors have been suggested to be chemoreceptive, mechanoreceptive, theromreceptive, or hygroreceptive in function [5,24]. However, there are no electrophysiological studies which support any of the potential functions, and their role in the biology of the insects that possess these receptors remains a mystery.

The rimmed pits found by Klein et al. [3] were not identified as sensory receptors but were hypothesized to secrete wax. The scape or pedicel was not sectioned, so any innervation pattern or cell type associated with these rimmed pits remains unknown [3]. These rimmed pores were only found on the scape and pedicel of *Magicicada* species. There are differences in the distribution pattern of these pores in the different species of *Magicicada* but they always are oriented in a single direction and are only found on the scape and pedicle. Variability may be a condition common to *Magicicada*, as Klein et al. [3] found differences in the distribution of sensilla in specimens of *M. cassinii* from different broods.

The cuticular spines found in *Beameria*, *Diceroprocta*, and *Pacarina* are similar to those found in the Asian cicadas [5]. Cuticular spines are found in greater phylogenetic diversity in Asia and Australia (nine genera, six tribes, three subfamilies, and two families) than they are in North America, where they were found in only three of the twelve genera examined and are restricted to a single tribe.

The lack of sexual dimorphism in the cicada antennae is not surprising. Species using acoustic or visual systems for mate attraction generally do not exhibit differences in antennal structure [19].

There are limited phylogenetic implications from the complete antennal morphology analysis. Although the basic segmental morphology of the antennae was consistent in all species studied, the elongated second flagellar segment in comparison to the first flagellar segment of *Neocicada* is unique to the genus in North America. This character state was also observed in the Asian Leptopsaltriini and the primitive Tettigarctidae Distant, 1905 species [5], so an elongated second flagellar segment appears to be a diagnostic character for Leptopsaltriini genera regardless of continental origin. Similarly, cuticular spines were absent in both North American and Asian genera of Leptopsaltriini and Tacuini but were present in Asian though not in North American Tibicinini. The cuticular spines were only found in Fidicinini genera in North America, a tribe composed completely of New World genera and very distantly related [10] to the Asian Cicadinae tribe (Dundubiini Distant, 1905) possessing cuticular spines.

The distribution of specific cicada antennal sensilla demonstrates mixed phylogenetic implications. All seven main types of antennal sensilla and cuticular spines are found in both the Asian species and the primitive Australian cicada [4,5]. However, there are some unique subtypes in the North American fauna, like the Asian fauna, that may be used to demonstrate evolutionary relationships.

There are unique receptors or subtypes that were found in a few genera or species of North American cicadas. Unique to the Cicadinae are the STrIII that were only found on the scape and pedicel of *Beameria* species (Fidicinini) and the grouping of several receptors in a single SCoII on the pedicel found in half of the species of *Diceroprocta* (Fidicinini) studied. Grouped SCoI were found centrally located on the first flagellar segment of Tibicininae species (Tibicinini and Platypediini). A single grouping was found in species of the genera *Tibicinoides* and *Okanagana* (Tibicinini), while seven groups, composed of two to five sensilla, were found in the single species of *Platypedia* (Platypediini) studied. The SCaII were found only on the scape, pedicel, and first flagellar segment of *Magicicada* species (Cicadettinae, Lamotialnini). Paired SSt or FO were found on distal flagellar segments of *Cacama* (Cicadinae, Tacuini) and *Tibicinoides* (Tibicininae, Tibicinini), which are distantly related taxa.

There is an evolutionary convergence in the reduced sensilla and the sensilla distribution pattern in grass-inhabiting species. The three species of *Beameria* (Cicadinae, Fidicinini) and *Okanagana synodica atrata* (Tibicininae, Tibicinini) have similar distributions of trichoid sensilla on the scape and pedicel and SCoI, SCoII, SSt, and FO sensilla on the first flagellar segment. The convergence of antennal morphology to *Beameria* species in *O. synodica atrata* is divergent from the other *Okanagana* species studied, which are arboreal [8]. Antennal sensilla appear to be useful for inferring habitat usage in grass-dwelling cicadas.

Cicada antennal sensory systems are much more complex than the antennae found in related Cicadomorpha Evans, 1946. Cercopidae Leach, 1815 have short antennae with a minimal number of basiconic, coeloconic, and trichoid sensilla restricted to portions of the scape, pedicel, and first flagellar segment, which has an expanded base [39,40,41], with campaniform sensilla found in some genera [40,41]. Species of Aphrophoridae Amyot & Audinet-Serville, 1843 have an antennal morphology with a similar construction to Cercopidae antennae, with the addition of a conical sensillum unique to the family [42]. The more distantly related Achiixiidae Muir, 1923, Kinnaridae Muir, 1925, and Cixiidae Spinola, 1839 of Fulgoromorpha Evans, 1946 exhibit greater divergence from the cicadas with microtrichia, cuticular denticles, plaque organs, scolopodia, styloconica, a basal flagellar process, and only the coeloconic sensilla shared with the cicadas [43,44,45], with one species of Delphacidae Leach, 1815 having additional unique sensilla and also sharing trichoid and campaniform sensilla with cicadas, although they are structurally distinct from the campaniform sensilla found in cicadas [46].

## 5. Conclusions

Cicada antennae from North America share the majority of sensilla reported in Asian cicadas [5]. The STrIII, SCaII, and groupings of SCoI or SCoII are unique to the North American genera that possess them. The first flagellar segment has the majority of non-hair-like sensilla classes present in all species studied. The presence of sensilla on more distal flagellar segments is related to the phylogeny of the species, with different types of receptors found in different genera or species. Continued investigations of the innervation patterns and electrophysiology of the sensilla will continue to inform the scientific community of the capabilities of cicada antennal organs.

## Figures and Tables

**Table 1:** Taxonomy of cicadas studied in this work. All species are classified in the Cicadidae Batsch, 1789. Taxa are ordered based on molecular phylogenies [10,15] so phylogenetic trends are easier to observe.

Subfamily	Tribe	Genus	Species
Cicadinae Batsch, 1789	Fidicinini Distant, 1905	*Diceroprocta* Stål, 1870	*D. apache* (Davis, 1921)
			*D. canescens* Davis, 1935
			*D. cinctifera cinctifera* (Uhler, 1892)
			*D. eugraphica* (Davis, 1916)
			*D. semicincta* (Davis, 1925)
			*D. swalei swalei* (Distant, 1904)
		*Beameria* Davis, 1934	*B. ansercollis* Sanborn & Heath, 2011
			*B. venosa* (Uhler, 1888)
			*B. wheeleri* Davis, 1934
		*Pacarina* Distant, 1905	*P. puella* Davis, 1923
	Tacuini Distant, 1904	*Neotibicen* Hill & Moulds, 2015	*N. pruinosus pruinosus* (Say, 1825)
			*N. superbus* (Fitch, 1855)
		*Hadoa* Moulds, 2015	*H. duryi* (Davis, 1917)
			*H. inaudita* (Davis, 1917)
		*Cacama* Distant, 1904	*C. collinaplaga* Sanborn & Heath, 2011
	Leptopsaltriini Moulton, 1923	*Neocicada* Kato, 1932	*N. chisos* (Davis, 1916)
Cicadettinae Buckton, 1890	Lamotialnini Boulard, 1976	*Magicicada* Davis, 1925	*M. cassinii* (Fisher, 1852)
			*M. septendecim* (Linnaeus, 1758)
			*M. septendecula* Alexander & Moore, 1962
			*M. neotredecim* Marshall & Cooley, 2000
Tibicininae Distant, 1904	Tibicinini Distant, 1905	*Tibicinoides* Distant, 1914	*T. hesperia* (Uhler, 1872)
			*T. rubrovenosa* (Davis, 1915)
			*T. utahensis* (Davis, 1919)
			*O. fumipennis* Davis, 1932
		*Okanagana* Distant, 1905	*O. magnifica* Davis, 1919
			*O. mariposa mariposa* Davis, 1915
			*O. synodica atrata* Dmitriev, 2020
			*O. gracilis gracilis* Davis, 1919
		*Okanagodes* Davis, 1919	*O. terlingua* Davis, 1932
	Platypediini Kato, 1932	*Platypedia* Uhler, 1888	*P. balli* Davis, 1936

## Data Availability

Original data available from the author on request.

## Abbreviations

The following abbreviations are used in this manuscript:

BBr	Böhm’s Bristle
CSp	Cuticular spine
FO	Foramen olfactorium
RP	Rimmed pore
SCaI	Sensillum campaniformium subclass I
SCaII	Sensillum campaniformium subclass II
SCav	Sensilla cavitata
SCh	Sensillum chaeticum
SCoI	Sensillum coeloconicum subclass I
SCoII	Sensillum coeloconicum subclass II
SSt	Sensillum styloconicum
STrI	Sensillum trichoidea subclass I
STrII	Sensillum trichoidea subclass II
STrIII	Sensillum trichoidea subclass III
